# New approaches suggest term and preterm human fetal membranes may have distinct biomechanical properties

**DOI:** 10.1038/s41598-022-09005-2

**Published:** 2022-03-24

**Authors:** Sudeshna Bhunia, Shaughn O’Brien, Yuting Ling, Zhihong Huang, Pensée Wu, Ying Yang

**Affiliations:** 1 School of Pharmacy and Bioengineering, Keele University, Stoke-on-Trent, ST4 7QB UK; 2 Academic Department of Obstetrics and Gynaecology, University Hospital of North Midlands, Stoke-on-Trent, ST4 6QG UK; 3 School of Science and Engineering, University of Dundee, Dundee, DD1 4HN UK; 4 School of Medicine, Keele University, Staffordshire, ST5 5BG UK

**Keywords:** Reproductive disorders, Mechanisms of disease, Cell biology, Diseases

## Abstract

Preterm prelabour rupture of membranes is the leading cause of preterm birth and its associated infant mortality and morbidity. However, its underlying mechanism remains unknown. We utilized two novel biomechanical assessment techniques, ball indentation and Optical Coherence Elastography (OCE), to compare the mechanical properties and behaviours of term (≥ 37 weeks) and preterm (33–36 weeks) human fetal membranes from ruptured and non-ruptured regions. We defined the expression levels of collagen, sulfated glycosaminoglycans (sGAG), matrix metalloproteinase (MMP-9, MMP-13), fibronectin, and interleukin-1β (IL-1β) within membranes by biochemical analysis, immunohistochemical staining and Western blotting, both with and without simulated fetal movement forces on membrane rupture with a new loading system. Preterm membranes showed greater heterogeneity in mechanical properties/behaviours between ruptured and non-ruptured regions compared with their term counterparts (displacement rate: 36% vs. 15%; modulus: 125% vs. 34%; thickness: 93% vs. 30%; collagen content: 98% vs. 29%; sGAG: 85% vs 25%). Furthermore, simulated fetal movement forces triggered higher MMP-9, MMP-13 and IL-1β expression in preterm than term membranes, while nifedipine attenuated the observed increases in expression. In conclusion, the distinct biomechanical profiles of term and preterm membranes and the abnormal biochemical expression and activation by external forces in preterm membranes may provide insights into mechanisms of preterm rupture of membranes.

## Introduction

Globally, preterm birth occurs in 10% of all live births with an estimated 15 million infants being born preterm per annum^1^. It causes significant morbidity such as neurodevelopmental impairment, learning difficulties and visual disorders^2,3^. Furthermore, the main cause of mortality for children aged under 5 arise from complications of preterm birth^4^. Sadly, management options of preterm birth are limited and include tocolysis, antibiotics, steroids for fetal lung maturity and rescue cerclage. Therefore, much research has focused on prevention of preterm birth. Preterm prelabour rupture of membranes (PPROM) is the leading cause of preterm birth occurring in 40% of premature neonates^5^. However, efforts in predicting and preventing PPROM have been hampered by poor understanding of the underlying pathophysiological mechanism.

Previous research has highlighted the importance of the biomechanical properties of fetal membranes in PPROM^6,7^, in addition to other maternal risk factors such as intrauterine infection, short cervical length, smoking and multiple pregnancy^8–10^. Fetal membranes undergoes cell proliferation and remodelling at microfractures to maintain structural integrity during fetal growth in pregnancy^11^. Aging and senescence of fetal membranes occurs with advancing gestation and contributes to term rupture of membranes and parturition^12^. The rupture site has altered morphology compared with other zones in fetal membranes, with increased connective tissue thickness but reduced tensile strength, and is located in fetal membranes overlying the cervix. This site also has decreased collagen content with increased matrix metalloproteinase (MMP) activity, apoptosis and inflammatory markers^13,14^. PPROM fetal membranes show signs of premature senescence activation^15^. Furthermore, infection, inflammation and oxidative stress have been shown to accelerate premature aging of fetal membranes with early inflammatory signalling causing PPROM^16^.

As animal models do not recapitulate human parturition adequately, various in vitro fetal membrane models have been used to study the process of fetal membrane rupture. The biomechanical properties of fetal membranes have been assessed using uniaxial tensile test, biaxial tensile test, burst or inflation test and puncture test^17^, which all have their own limitations. Uniaxial tensile test does not mimic the loading condition in vivo, whereas bi-axial tensile test is not suitable for testing chorioamniotic membranes which are thin and slippery. The burst or inflation test requires large sample sizes, while the locally applied force to displace the sample in the puncture test fails to match the loading conditions found in vivo^18^. In the present study, we adapted two novel non-destructive mechanical testing techniques (ball indentation to test the displacement behaviour and Optic Coherence Elastography (OCE) to test elastic modulus) to demonstrate the differences in biomechanical properties between term and preterm human fetal membranes. We also developed a new cyclical loading system to investigate the effects of repeated external forces on fetal membrane properties between term and preterm, and its potential to mitigate by nifedipine, a commonly used tocolytic drug in preterm birth management.

## Results

### Term and preterm fetal membranes have distinct mechanical properties

**Figure 1:**
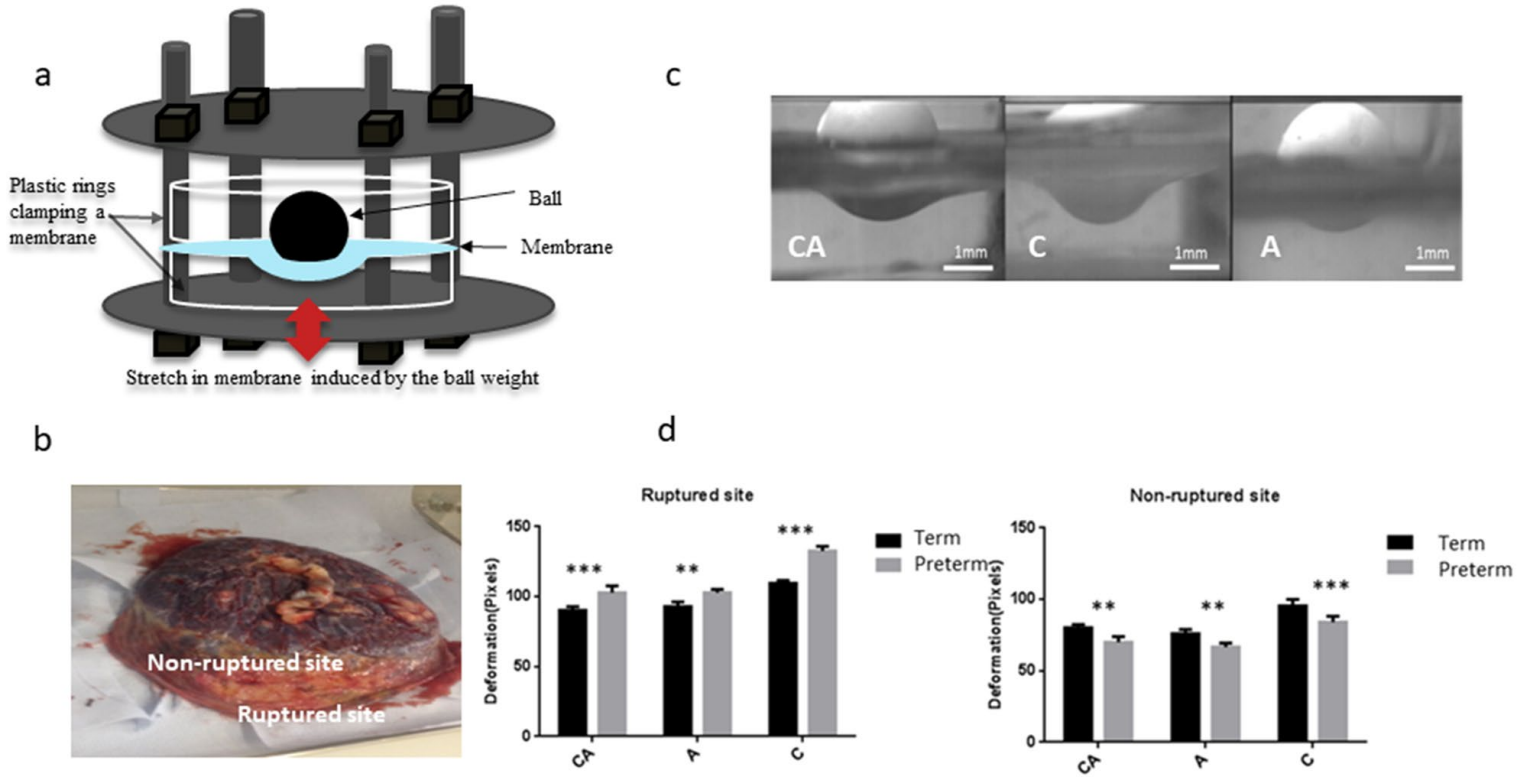
Ball indentation test of mechanical behaviours of fetal membranes. (**a**) Schematic drawing of ball indentation working principle. (**b**) The image demonstrating the locations of the rupture site (tear area) and non-ruptured site (area far from rupture site or close to umbilical cord). (**c**) Representative displacement images of the membranes following loading for 4 h. *CA* chorioamnion, *C* chorion and *A* amnion. Scale bar is 1 mm. (**d**) Quantification of the displacement following loading for 4 h for ruptured and non-ruptured membranes between preterm and term samples. (n = 4). Data are presented as mean ± SD. **p < 0.01, ***p < 0.001.

The ball indentation setting developed in our group^19^ can measure displacement behaviour of thin membranes conveniently. In this study, we used the ball indentation test to study the displacement behaviours of fetal membranes (Fig. 1a–d). This test aims to simulate the pressures exerted on fetal membranes within the gravid uterus from fetal weight and amniotic fluid, particularly to reproduce the in vivo multiaxial loading state of the membrane over the cervix. We demonstrated significant displacement behaviour difference not only between fetal membranes from term and preterm deliveries, but also between ruptured and non-ruptured regions within the same membrane, as well as between amnion alone, chorion alone and chorioamnion combined (Table 1).

**Table 1:** The mechanical properties and key ECM molecules at ruptured and non-ruptured site of term and preterm membranes. The difference ratios are calculated relative to rupture site values. *A* amnion, *C* chorion, *CA* chorioamnion, *sGAG* sulphated glycosaminoglycans.

	Full term				Preterm			
	Non-ruptured	Ruptured	*p* value	Difference ratio (%)	Non-ruptured	Ruptured	*p *value	Difference ratio (%)
Displacement (CA) (pixels)	80	94	0.001	14.9	71	110	0.0004	35.5
Displacement (A) (pixels)	80	95	0.0003	15.8	71	103	0.0001	31.1
Displacement (C) (pixels)	97	110	0.001	11.8	88	135	0.0002	34.8
Modulus (kPa)	489	365	0.01	34	713	317	0.0001	125
Thickness (mm)	0.39	0.3	0.008	30	0.77	0.4	0.0001	92.5
Total collagen (µg/µl)	6.50	5.02	0.0003	29.4	8.84	4.46	0.0001	97.9
Total GAG (µg/µl)	21.7	17.4	0.006	24.7	27.7	14.9	0.001	84.9

Maximum displacement was detected in chorion compared with the other 2 types of membranes (Fig. 1c). There was minimal displacement in amnion after the ball loading for 3–4 h and slight displacement after 6–8 h with immediate recovery after the ball was removed. Chorion maintained displacement for hours with some exhibiting permanent displacement. Increased displacement was observed in ruptured sites compared with non-ruptured sites of fetal membranes (Table 1). Interestingly, the ruptured sites exhibited greater displacement while the non-ruptured sites showed little displacement when preterm and term fetal membranes were compared with 14.9% difference ratio in term and 35.5% in preterm membranes (Table 1).

**Figure 2:**
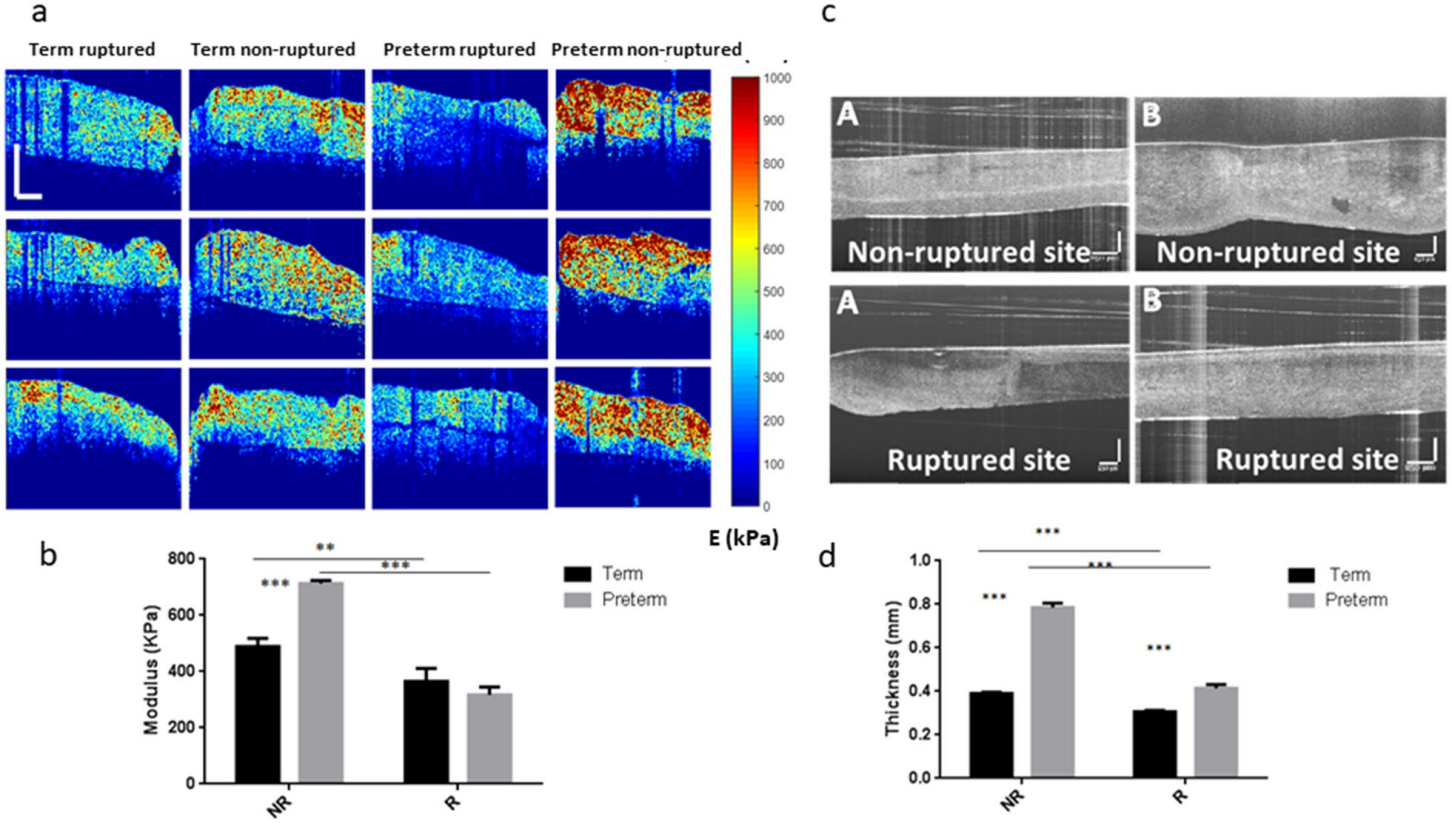
OCE and OCT tests of mechanical properties of fetal membranes. (**a**) Representative OCE images showing different stiffness between preterm and term samples in non-ruptured and ruptured sites (n = 3). Colour scale bar indicates the stiffness with unit of kPa. Scale bar is 600 µm. (**b**) Quantitative analysis of the modulus in membranes of OCE results. (**c**) Representative OCT images showing the variation of membrane thickness between term and preterm samples in non-ruptured and ruptured sites. Scale bar is 250 µm. (A) term and (B) preterm sample. (**d**) Quantitative analysis of the thickness of preterm and term membranes (R: ruptured and NR: non-ruptured site). Data represents in mean ± SD. (n = 4). **p ≤ 0.01, ***p ≤ 0.001.

Figure 2a shows representative stiffness mapping images from OCE measurements of preterm and term fetal membranes in ruptured and non-ruptured sites. The stiffness of the non-ruptured site was higher than the ruptured site in general. However, the non-ruptured site of preterm membrane showed a large difference in comparison with the ruptured site (713 ± 11 kPa vs. 317 ± 29 kPa, *p* = 0.001, with 125% difference ratio, Fig. 2b, Table 1). In contrast, little difference in stiffness was observed between the non-ruptured and ruptured sites in term membranes (489 ± 29 kPa vs. 365 ± 46 kPa, *p* = 0.01, with 34% difference ratio, Table 1). Furthermore, preterm membranes exhibited greater stiffness than term membranes in non-ruptured site only: 713 kPa vs 489 kPa, *p* = 0.0001, not in ruptured site: 317 kPa vs 365 kPa, *p* = 0.1.

Fetal membrane thickness of different groups of membranes measured by Optical Coherence Tomography (OCT) showed that non-ruptured sites were thicker than ruptured sites (Fig. 2c). Similar to the OCE findings, there was a vast difference between the non-ruptured and ruptured sites (0.77 mm vs. 0.40 mm, *p* = 0.0001) in preterm membranes, while the thickness in both sites (0.39 mm vs. 0.30 mm, *p* = 0.008) were only slightly different in term samples (Fig. 2d, Table 1). In addition, preterm membranes were significantly thicker than term membranes within each site (non-ruptured *p* < 0.01, ruptured *p* < 0.001).

### Expression of biochemical markers correlated with mechanical properties of fetal membranes

**Figure 3:**
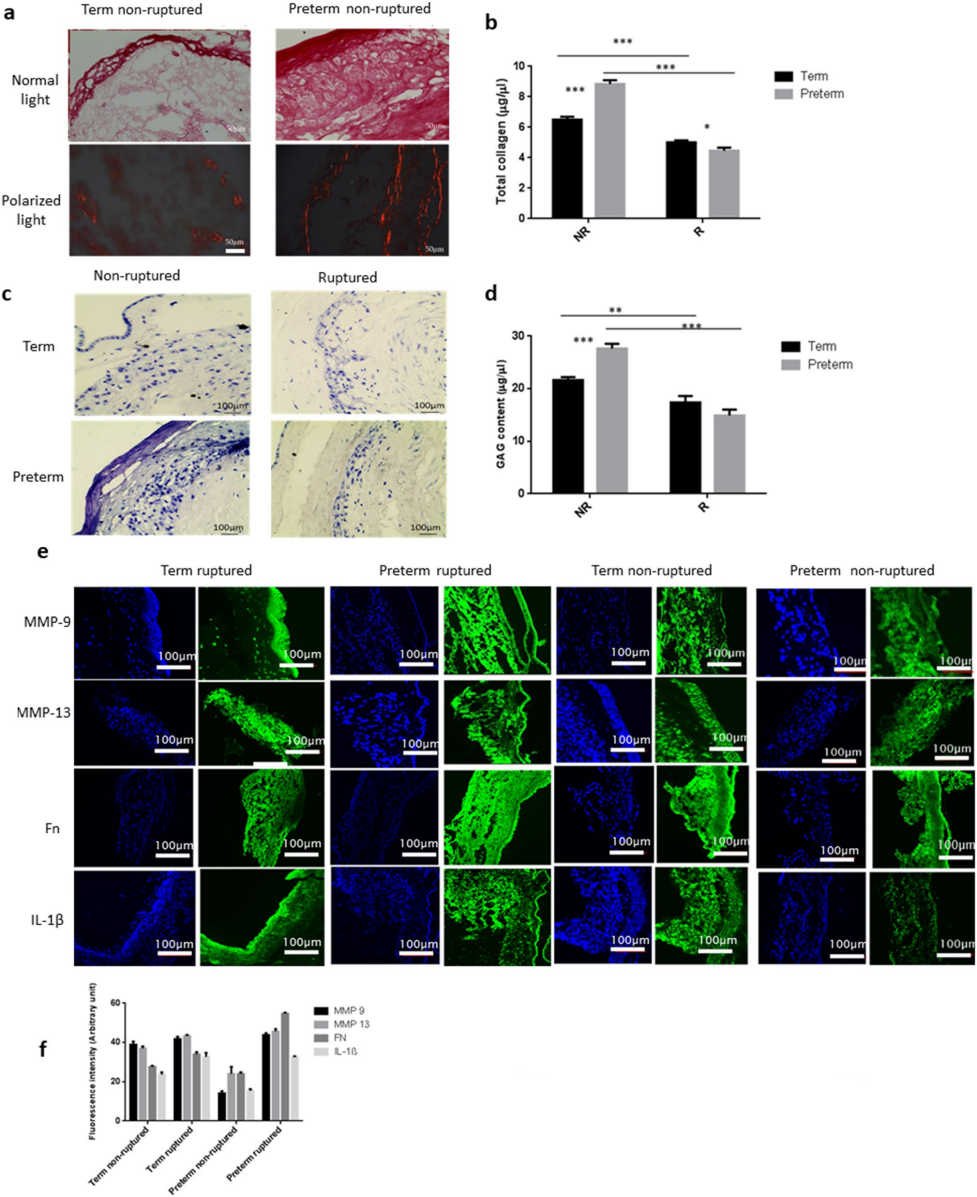
Histochemical and immunohistochemical analysis of key proteins and ECM molecules in the fetal membranes. (**a**) Pico Sirius Red staining of collagen fibres (n = 3). (**b**) Total collagen content (n = 3). NR, non-ruptured site. R, ruptured site. (**c**) Toluidine blue staining for sGAG (n = 3). (**d**) 1,9-dimethylmethylene blue (DMMB) assay for sGAG content. Data are presented as mean ± SD (n = 3). **p < 0.01. ***p < 0.001. (**e**) Representative images showing biomarker expression (green) with counter staining by DAPI (blue). (**f**) Quantitative analyses of the immunostaining intensity. *Fn* fibronectin, *IL* interleukin, *MMP* metalloproteinase. Scale bar is 100 µm.

Next, we analysed multiple key proteins and biomarkers expression patterns of preterm and term fetal membranes in correlation with their mechanical properties. Pico Sirius Red staining revealed that collagen fibres were thicker, aligned more compactly and stained more intensely in non-ruptured sites of preterm compared with term membranes (Fig. 3a). We assessed total collagen quantity using hydroxyproline assays and showed that non-ruptured had significantly higher quantity of collagen than ruptured sites, with a greater difference in preterm (8.84 vs. 4.46 μg/μl, *p* = 0.0001) than term fetal membranes (6.50 μg/μl vs. 5.02 μg/μl, *p* = 0.0003) (Fig. 3b, Table 1).

Total sulfated glycosaminoglycans (sGAG) level in fetal membranes was estimated by toluidine blue staining and 1,9-dimethylmethylene blue (DMMB) assay (Fig. 3c,d). Toluidine blue staining showed non-ruptured sites had higher sGAG expression than ruptured sites in both preterm and term samples (Fig. 3c,d). The most prominent staining was observed in the spongy layer, an interface between the amnion and chorion, of fetal membranes, which was more clearly displayed in non-ruptured site of preterm samples. Of note, ruptured sites within preterm membranes stained more intensely than its counterpart in term membranes. The DMMB assay findings were consistent with toluidine blue staining data, showing significant differences between non-ruptured and ruptured sites in preterm (85.8%) and term (24.6%) membranes (Fig. 3d, Table 1).

**Figure 4:**
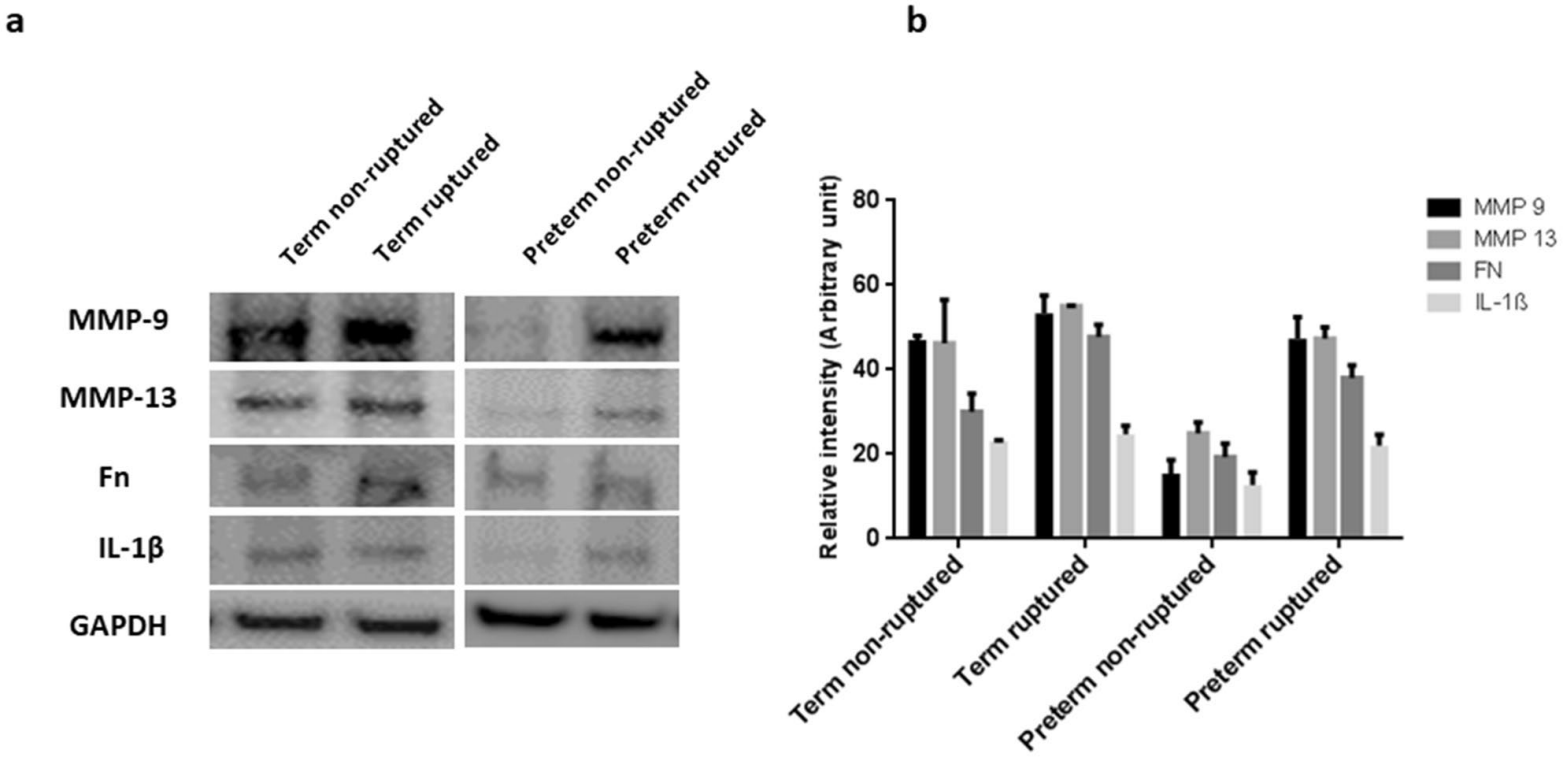
Western blotting analysis of key protein expression. (**a**) Representative images. (**b**) Quantitative analysis of the protein bands with normalization of the proteins to GAPDH (n = 3). The bands were cropped, grouped and processed in parallel from different parts of the same gel/same experiment for better visualization and comparison. The samples derive from the same experiment and that gels/blots were processed in parallel. Data are presented as mean ± SD. *Fn* fibronectin, *IL* interleukin, *MMP* metalloproteinase. Full-length blots/gels images with different exposures are presented in Supplementary Fig. 4.

We then assessed the expression of selected metalloproteinase and proteins which control extracellular matrix metabolism using immunohistological staining. Overall, non-ruptured sites contained lower levels of MMP-9, MMP-13, fibronectin and interleukin-1β (IL-1β) compared with ruptured sites, with a greater difference in protein expression observed in preterm compared with term membranes (Fig. 3e,f). We cross-validated these results using Western blotting and confirmed that the expression patterns of MMP-9, MMP-13, fibronectin and IL-1β were consistent with the immunohistological staining findings, with greater disparity between non-ruptured and rupture sites noted in preterm than term membranes (Fig. 4).

### Term and preterm fetal membranes responded differently to external forces

**Figure 5:**
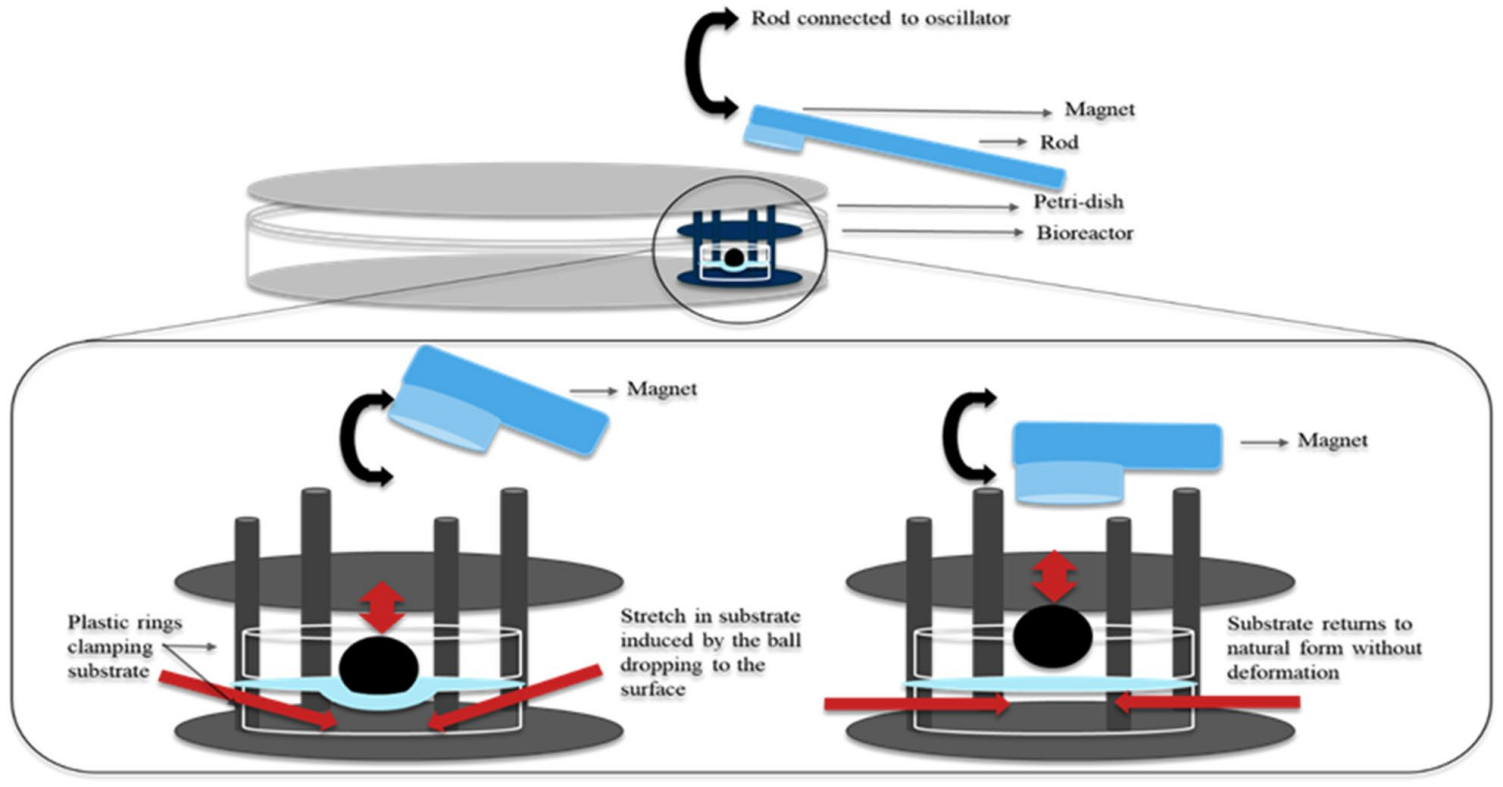
Diagram showing the external loading system to exert cyclical force on fetal membranes. A moving rod attaching with a magnet driven by a rocker lifted and dropped the metallic ball to fetal membranes alternately while the whole loading system was placed in an incubator at 37 °C and 95% CO_2_.

**Figure 6:**
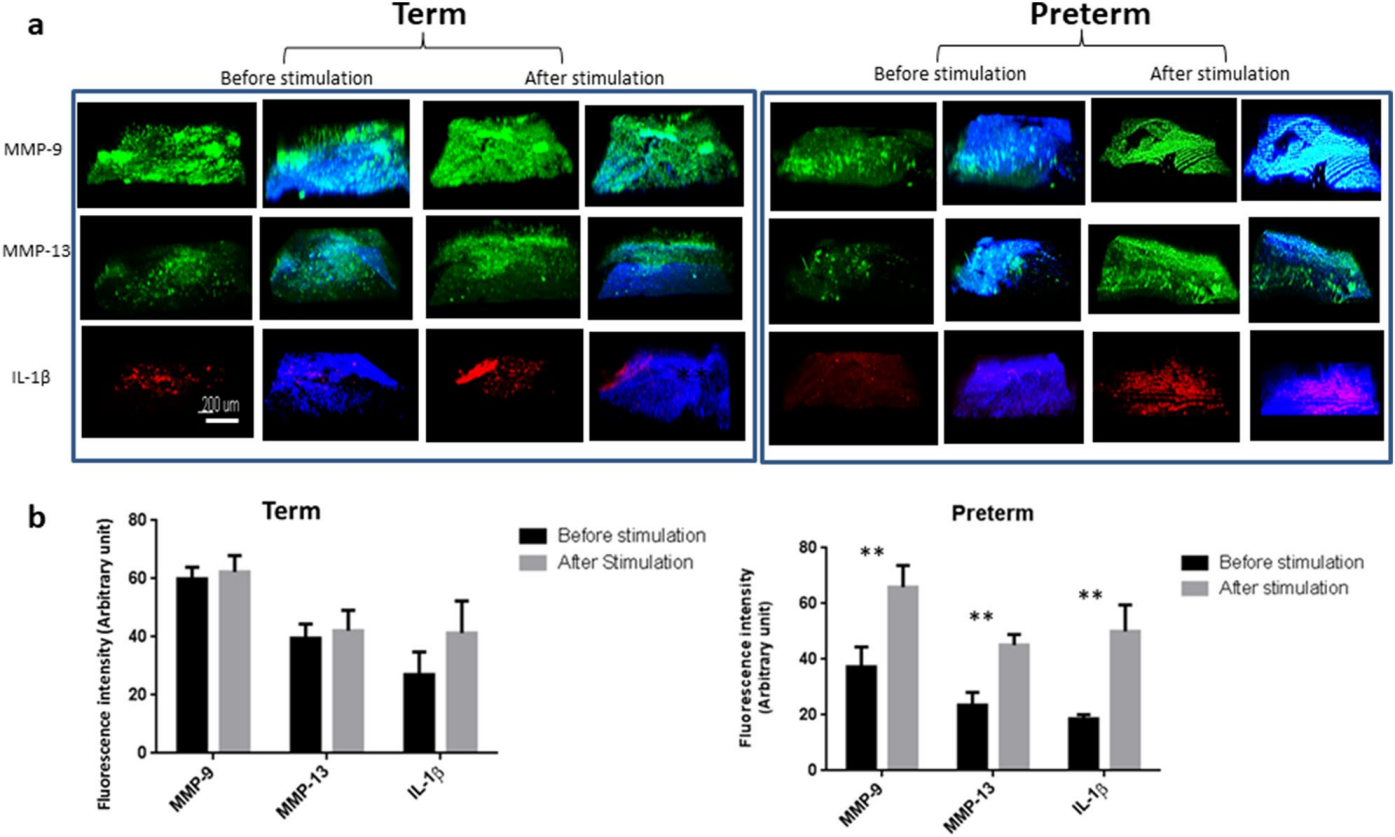

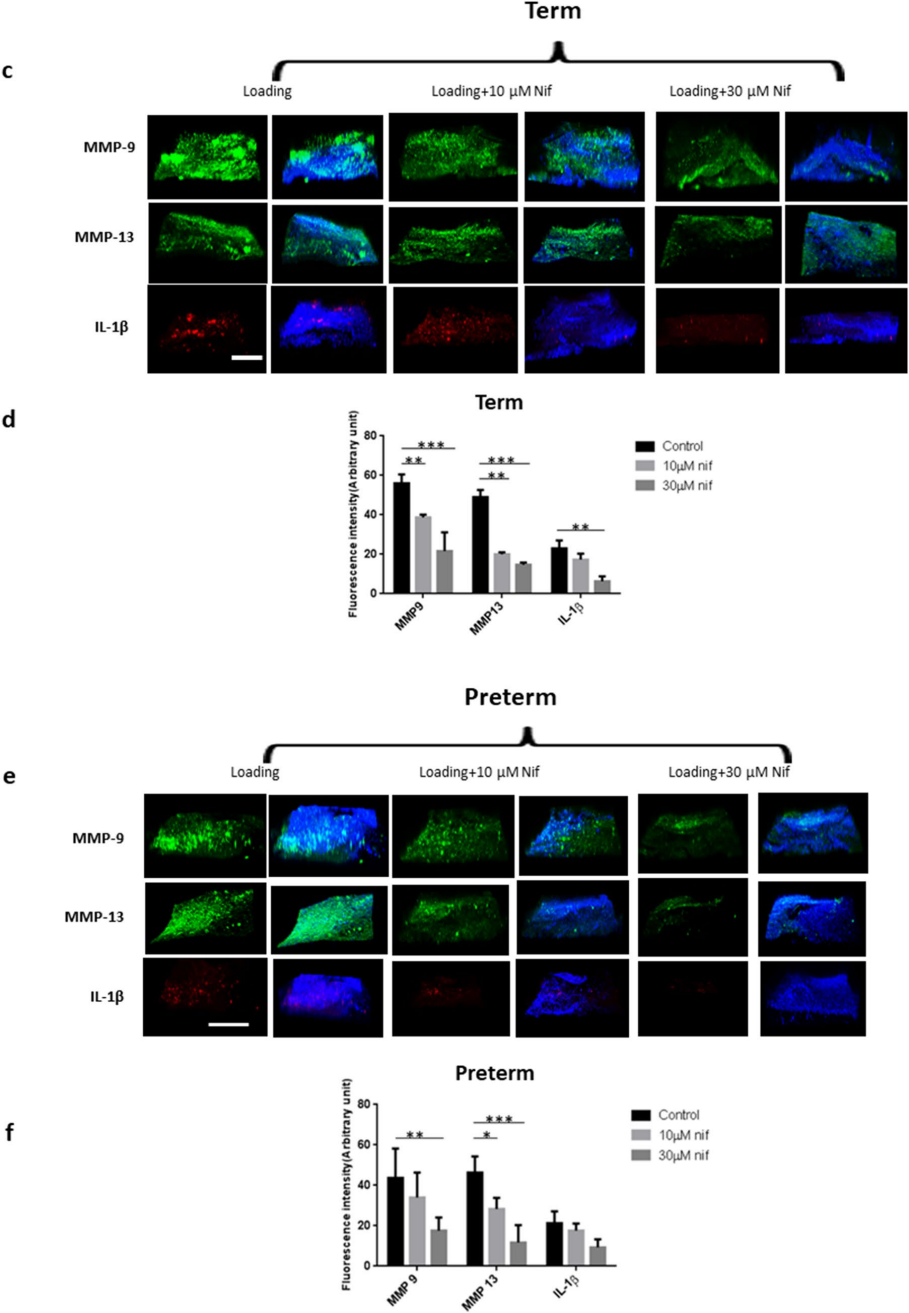
Immunohistochemical staining showing the effect of mechanical force on the change of key biomarkers expression with and without addition of nifedipine (nif). (**a**) Representative images showing biomarker expression, MMP-9 (green), MMP-13 (green) and IL-1β (red), with counter staining by DAPI (blue) merged, before and after the 3 h cyclic loading plus 4 h post-loading incubation without addition of nifedipine. (**b**) Quantitative analyses of the immunostaining intensity of images in (**a**). (**c**) Representative images showing biomarker expression, MMP-9 (green), MMP-13 (green) and IL-1β (red), with counter staining by DAPI (blue) merged, before and after the 3 h cyclic loading plus 4 h post-loading incubation with addition of 10, and 30 µM nifedipine for term membranes. The loaded samples without addition of nifedipine were used as control. (**d**) Quantitative analyses of the immunostaining intensity of the images in (**c**). (**e**) Representative images showing biomarker expression, MMP-9 (green), MMP-13 (green) and IL-1β (red), with counter staining by DAPI (blue) merged, before and after the 3 h cyclic loading plus 4 h post-loading incubation with addition of 10, and 30 µM nifedipine for preterm membranes. The loaded samples without addition of nifedipine was used as control for preterm samples. (**f**) Quantitative analyses of immunostaining intensity of the images in (**e**). *Fn* fibronectin, *IL* interleukin, *MMP* metalloproteinase. Scale bar is 200 µm.

**Figure 7:**
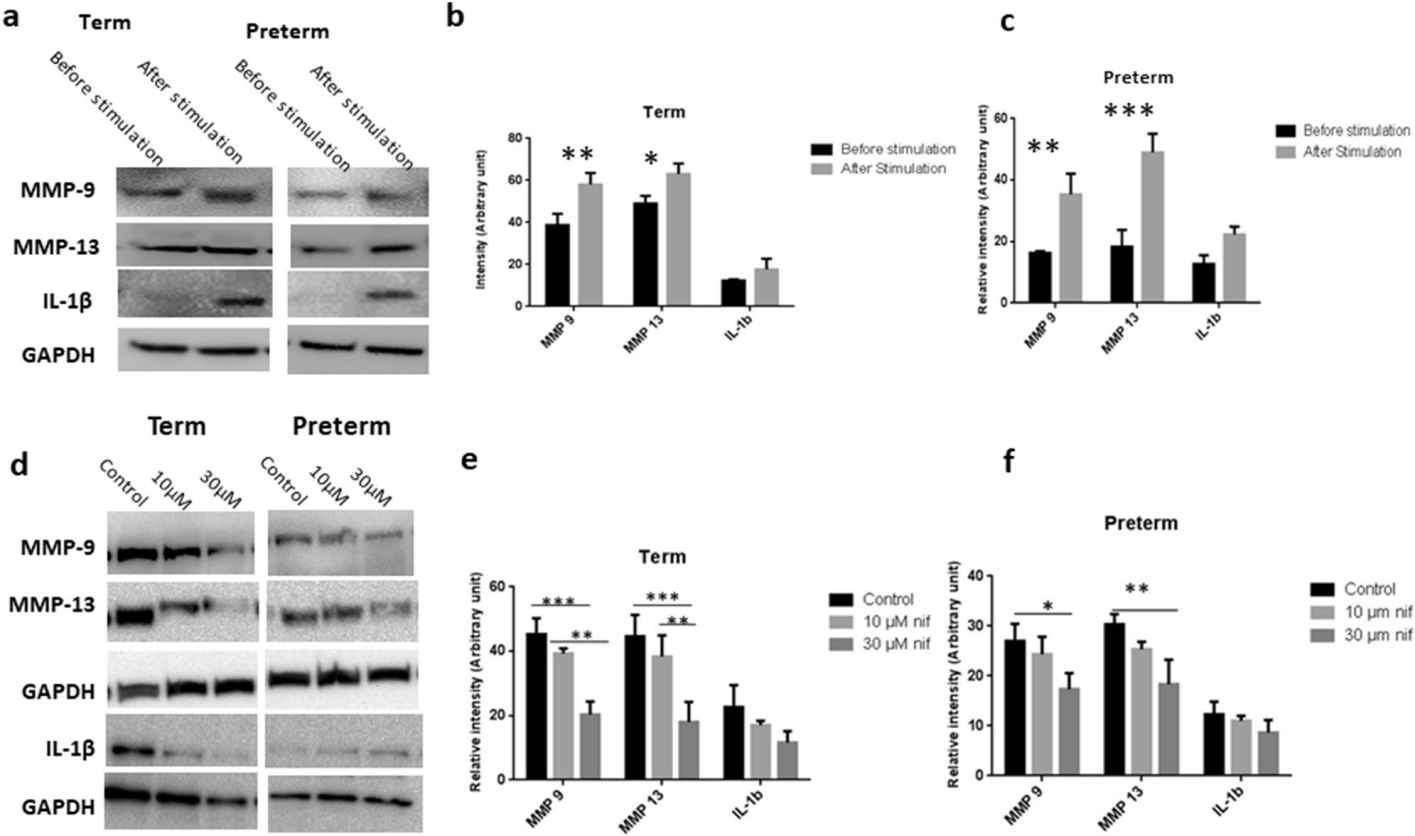
Effect of cyclical loading on key biomarker expression analysed by Western blotting with and without addition of nifedipine. The bands were cropped, grouped and processed in parallel from different parts of the same gel for better visualization and comparison. Before and after cyclical loading shown by (**a**) representative images and quantitative analyses of protein bands with normalization of the proteins to GAPDH in (**b**) term and (**c**) preterm membranes. With and without addition of nifedipine (nif) during loading illustrated by (**d**) representative images and quantitative analyses in (**e**) term and (**f**) preterm membranes. The bands were cropped from different parts of the same gel and grouped for better visualization and comparison. Data are presented as mean ± SD (n = 3). *IL* interleukin, *MMP* metalloproteinase, *nif* nifedipine. *p < 0.05. **p < 0.01. ***p < 0.001. Full-length blots/gels images *with different* exposures are presented in Supplementary Fig. 5.

The ball indentation system exerts radial directional stretch to membranes conveniently and biomimically. We altered the static loading from the ball into a cyclical loading system by alternately lifting and dropping the metallic ball to the membranes with a magnetic moving rod (Fig. 5). We hypothesized that this cyclical loading system mimics intrauterine fetal movement forces exerted on the membranes and investigated expression of MMP-9, MMP-13 and IL-1β in non-ruptured fetal membranes following cyclical stimulation. Term membranes showed higher expression of MMP-9 and MMP-13 than preterm counterparts prior to stimulation. Following cyclical loading, MMP-9 and MMP-13 levels increased in both fetal membranes, with greater increase in preterm than term membranes. For IL-1β, we found minimal expression before loading and increased expression after cyclical stimulation in both fetal membranes, with a greater increase also observed in preterm membranes following stimulation (Fig. 6). Western blotting analysis further validated the immunostaining findings (Fig. 7). Taken together, preterm samples showed higher sensitivity to external forces than term membranes.

### Nifedipine attenuates the effects of external forces on fetal membranes

To assess the effect of the calcium channel blocker nifedipine, a tocolytic agent used in clinical practice, on key protein expressions, fetal membranes were treated with nifedipine during cyclical loading. Using immunohistological staining and Western blotting assays, we found nifedipine attenuated the increased expression of MMP-9, MMP-13 and IL-1β following cyclical loading in both preterm and term membranes at therapeutic concentrations (10–30 μM) (Fig. 6). There was a dose–response relationship with the degree of attenuation of protein expression levels being inversely proportional to increasing concentrations of nifedipine.

## Discussion

The fetal membrane is a strong and load-bearing tissue, its integrity secures the growth of the fetus to full term. As fetal membrane rupture is a prerequisite for birth, an understanding of the biomechanical properties of fetal membranes may help us to identify potential causes of PPROM. In this study, we applied two novel mechanical testing modalities to assess fetal membranes after term and preterm rupture of membranes in parallel. We provided evidence that greater heterogeneity of mechanical and biochemical properties existed within preterm compared with term fetal membranes. For the first time, we showed that preterm membranes had higher sensitivity to external force stimulation with increased biomarker expression than term membranes.

Prior research has been conducted on biomechanical properties of the fetal membranes with varying mechanical testing methodologies described in the literature. However, most test modalities are not appropriate for fetal membrane studies and have resulted in inconsistent conclusions. The widely reported method of the Com-Ten Ball-Burst compression testing equipment uses a motor driven plunger to deform fetal membranes radially^20^. Although it has generated consistent evidence of a zone of altered morphology in fetal membranes overlying the cervix, it has not been used to study the very weak site within fetal membranes where rupture has spontaneously occurred. Most importantly, none of the current testing methods can be used to investigate the alteration in biomarker expression in response to external loading of fetal membranes.

The ball indentation technique used in this study offers a simple and reliable method to obtain displacement behaviours of fetal membranes with prolonged incubation time under physiological conditions. Fetal membranes are viscoelastic materials. The resistance to displacement under constant load (weight of ball), reflects the integrity and degradation state of fetal membranes. The lateral displacement images conveniently recorded the dynamic changes of the membranes and allows comparison of samples from the same or different placenta. Importantly, the test conditions closely mimicked the in vivo environment as all tests were conducted in an incubator set at body temperature while the weight of the ball exerted constant stretching force to the membranes in radial directions, which simulates fetal weight and amniotic fluid loading on fetal membranes.

The OCE technique examines the stiffness of the membranes without requiring clamping, which eliminates artefactual changes. The current OCE setting uses phase sensitive optical coherence tomography (PhS-OCT) combined with a magnetic shaker, which is driven by a sine-wave modulated signal to generate vibration to the samples. The PhS-OCT with spectral-domain configuration was utilized to detect the vibration signal of tested samples. The fetal membrane samples have a layered structure. During our OCE scanning process, the fetal membrane was placed on the homogenous 2% agar phantom to form a composite layered sample with the known Young’s modulus of the agar phantom as a stress sensor^21–23^. The underlying working principle to obtain the modulus is that when the vibration stimulation is applied to a two-layered sample, a uniform stress field can be assumed to apply to both layers. The assumption of uniform stress presented across the bi-layer sample area has been verified by Kennedy et al.^22^. A bi-layer phantom with modulus contrast 37:1 was utilised in both experiment and simulation. It was revealed by OCE that the stress distribution of the bi-layer sample was uniform without stress concentrations or strain artefacts apparent at the interface of the two materials. The observed strain contrast was able to match the module contrast between the layers. In continuum mechanics, if the bi-layers have homogenous material properties, and the interface of the two layers have no stress concentrations or stress gradients or strain artefacts, the external force applied to the bi-layers will be equal to internal forces; and each layer will produce different strain toward the exerted force if they have different stiffness^22,23^. Thus, under the same stress (σ1 ≈ σ2) in a layered material, the ratio of Young’s modulus in layer 1 and 2 is equal to the inverse ratio of strain ε1 and ε2. The local strain is determined from detected tissue displacement caused by the vibration amplitude which can be calculated by the fast Fourier transform (FFT) of the phase difference [FFT (Δϕ)]^21–23^. Therefore, using compound samples with the tested sample overlying a reference agar layer with known mechanical properties, the Young’s modulus of the test sample can be quantified by the following equation^23^.

$$\frac{{E}_{1}}{{E}_{2}}=\frac{{\sigma }_{1}/{\varepsilon }_{1}}{{\sigma }_{2}/{\varepsilon }_{2}}=\frac{{\sigma }_{1}{\varepsilon }_{2}}{{\sigma }_{2}{\varepsilon }_{1}}\approx \frac{{\varepsilon }_{2}}{{\varepsilon }_{1}},$$

where E is Young’s modulus, σ is stress, ε is strain, 1, 2 are sample and agar. The Young’s modulus of the single-layer agar phantom has been investigated in the previous study^21^ in which with the measured average phase velocity (7.55 ± 1.09 m/s) for a 2% agar phantom of the shaker-induced surface wave, the Young’s modulus of the 2% agar phantom has been calculated using the Eq. (1) below.

1 $$C_{{\text{R}}} = \frac{0.87 + 1.12v}{{1 + v}}\left( {\frac{E}{2\rho (1 + v)}} \right)^{1/2},$$

where C_R_ is the surface wave phase velocity, E is the Young’s modulus, ν is the Poisson’s ratio and ρ is the density of the material. We used 0.5 as Poisson’s ratio based on previous research^24^ for soft tissues which are incompressible due to the high content of water. This gives a calculated Young’s modulus of 193 ± 4.01 kPa for 2% agar phantom^21^.

It was interesting to find that chorion had maximum displacement, much larger than amnion. Though amnion is much thinner (20% of whole membrane thickness) than chorion, it has been found to be much stronger in comparison to chorion^25^. In a time dependent study, Mauri et al. investigated the microstructural response of amnion^26^. They found reversible short term displacement of the membrane as a result of water outflow from the collagenous network. Some studies postulated that amnion is affected more during labour compared to chorion, whilst at the same time being stronger and less likely to undergo displacement than chorion^25^. It is unclear whether the thickness we measured is related to cellularity or cellular level changes as we did not assess the relationship between cellular content and the thickness of fetal membranes in this study.

We found membranes from ruptured areas showed larger displacement and lower modulus compared with those from sites distant to ruptured areas. This complements the description of the rupture site containing a zone of altered morphology in the literature^13,20,27^. Furthermore, we are the first to show in parallel that the heterogeneity in biomechanical and biochemical properties between non-ruptured and ruptured sites is greater in preterm than term membranes. We found that the difference in modulus between sites was 125% in preterm membranes, compared with 34% in term membranes which is close to prior research on term fetal membranes demonstrating a 55% difference in rupture strength^20^.

Fetal membrane is a complex tissue. The mechanical behaviour of fetal membrane is variable and dependant on several factors, such as gestation, mode of delivery, location within the membrane (ruptured and non-ruptured), separation between amnion and chorion, collection method and many others. Therefore, the testing methods and the experiment setup between different researchers can generate diverse results for biomechanical properties of the fetal membranes. For instance, the modulus value from tensile test of term fresh chorioamniotic membrane from Oxlund et al.^28^ and Chowdhury et al.^29^ were 15 MPa and 4.9–7.5 MPa respectively, which is higher than the values from OCE in this study. However, the outcomes of the mechanical properties of fetal membranes between different membrane types, for example ruptured versus non-ruptured location, allows for comparison within a particular study.

We also observed a disparity between preterm and term membranes in thickness. The reported thickness of fetal membranes by histological analysis was consistent with our outcome for term samples; for example in the cervical area, 298 ± 60 μm, versus the mid-zone area, 327 ± 64 μm^30^. Ultrasonography is the most frequent imaging modality to measure fetal membrane thickness. Severi et al.^31^ reported that preterm had greater fetal membrane thickness, 1.67 ± 0.27 mm, versus term membrane, 1.14 ± 0.30 mm. Our study showed the same trend, however the absolute thickness values are different.

From an engineering point of view, a large mechanical property difference in a material leads to stress concentration when subjected to external stress, where the stress in some sites is significantly greater than their surrounding regions^32,33^. Hence, spatial heterogeneity and material anisotropy (inhomogeneity) can induce cracks in the material. Microfractures that are greater in number and dimension have been observed in membranes from PPROM, compared with membranes from term labour^34^. Therefore, we postulate that the high mechanical property heterogeneity in preterm membranes may be a potential factor for the observed increase in microfractures and premature aging within membranes from PPROM.

Findings of the biochemical analyses echoed those from mechanical tests. We show for the first time, the difference in MMP-9, MMP-13, fibronectin and IL-1β protein expression between non-ruptured and ruptured sites was greater in preterm than term membranes. Our biomarker studies showing higher expression of these proteins in ruptured than non-ruptured sites are in keeping with the literature. High MMP expression level linked to collagen content. Hampson et al.^35^ compared collagen samples from 15 preterm prelabour rupture of the amniotic membranes (PPROM) and 25 delivering at term membranes by interrupted gel electrophoresis. It was found that collagens were decreased in PPROM relative to controls. Our study took one step further to analyse the sub-difference of collagen content between the rupture and non-rupture region of term and preterm membranes. The rupture site of preterm membrane had lower collagen content than term membrane in both region, which is consistent with previous study. The higher collagen content in non-rupture region of preterm membrane supports our hypothesis that preterm membranes have heterogeneity properties. It was also noted that all of the protein expression levels in ruptured sites of preterm samples reached values approximating that in term samples which had similar expression levels between ruptured and non-ruptured sites.

MMP‐9 expression is increased in membranes from term and preterm labours compared with those without labour^36,37^. Higher MMP-13 levels have been observed in vaginal fluid washings from women with PPROM compared to women with healthy pregnancies at the same gestation^38^. Previous studies show that IL-1β increase in term and preterm labour where it activates MMP-9 and induces apoptosis^39–41^. Furthermore, IL-1β causes biomarker changes characteristic of the zone of altered morphology seen in rupture sites^42^. We recognize that immunostaining cannot distinguish between active and inactive forms of MMPs. However, we found co-localisation of high MMP-9 and MMP-13 levels along with low total collagen concentration and poor organization of collagen fibres as functional studies in parallel experiments from the same membrane type and location. This suggests that the measured MMPs were in active form and supported by a previous study by Dahlen et al.^43^.

Fibronectin is an extracellular matrix glycoprotein located between chorionic membrane and uterine decidua, and is used clinically as a marker of spontaneous preterm labour. It has been shown to activate MMP-9 in response to inflammation as well as participate actively in up-regulating MMP-9 and MMP-13 mRNA^44,45^. Our study clearly demonstrate that preterm samples express abnormally high biomarkers following rupture, which may have triggered premature ageing of the membranes.

It has been postulated that the force from membrane distension as the pregnancy progresses, not only acts as a source of cellular stress through physical strain, but may also provide the link between biochemical and mechanical changes in parturition. Mechanotransduction studies on fetal membranes show that stretch force induces pro-inflammatory cytokine production, such as IL-1β, and regulates apoptosis^46–48^. Taken together, these data illustrate how the heterogeneity in mechanical properties between ruptured and non-ruptured sites in preterm and term membranes translates to the distinct biochemical and inflammatory signature that breaks down extracellular matrix through MMP production and predisposes to PPROM.

Using our model that simulates the effects of cyclical intrauterine fetal movement forces on fetal membranes, we found that preterm membranes showed higher sensitivity to external forces with a greater increase in expression levels of IL-1β, MMP-9 and MMP-13 following loading stimulation, compared with term membranes. One previous study utilised a bioreactor to model cyclical tensile strain on term fetal membranes in a dumbbell shaped strip and showed increased total MMP activity following cyclical stimulation^48^. It is worth noting under normal physiological settings, term membranes experience greater loading forces compared with preterm membranes due to fetal growth with increasing gestation.

As well as being a tocolytic agent used in the clinical management of preterm labour, nifedipine is a well-known calcium-channel blocking agent. In this study, we used nifedipine to validate the role of calcium channels in the mechanotransduction pathway. We found nifedipine, a calcium channel blocker, considerably attenuated the increased expression of MMP-9 and MMP-13 following cyclical loading. A stretch-activated channel can directly activate ion influx^49^, which may result in an influx of calcium ions that promote the synthesis of pro-inflammatory cytokines and matrix degrading proteins^50^. Calcium channel blockers have been shown to inhibit tissue remodelling by reducing oxidative stress and MMP production^51^, suppress nitric oxide synthesis mRNA, ROS and TNF-α production, and reduce MMP-9 levels without affecting TIMP-1 or MMP-2 levels^52^. Therefore, the tocolytic effects of nifedipine may involve downregulation of inflammatory cytokines and MMPs.

There are limitations to our study. It is difficult to fully reproduce the in-vivo loading conditions of the fetal membranes as the uterine, abdominal and pelvic floor muscles provide external support which limit the exposure of fetal membranes to internal pressure forces. Therefore, the overall deformation of the fetal membranes is also determined by the compliance of these muscles which differ between individuals. Although the ball indentation test may be used to study the displacement behaviour of the tissue over the cervix, the identification of this exact tissue area from ruptured placenta is difficult. The modulus from this OCE measurement is obtained from a specific strain rate determined by vibration frequency. The absolute modulus may be different from conventional quasi-static modulus which is obtained at a low strain rate. Another limitation of our study arises from the vaginal delivery specimens having undergone the labour process. As labour can lead to pre-mechanical conditioning of the membranes, there are additional variability between the different fetal membrane groups under comparison, which we could not control for as each labour is intrinsically different.

## Conclusion

Using new mechanical testing approaches for fetal membranes, our study demonstrates the distinct biomechanical signatures between preterm and term rupture of membranes. We show that preterm fetal membranes have higher sensitivity to external forces than term membranes, while calcium channel blockers attenuate these effects via an inverse dose–response relationship.

Our findings add to the growing body of evidence suggesting that PPROM occurs because of premature senescence activation from oxidative stress and inflammatory processes. We illustrate a potential mechanism for PPROM, where once premature aging occurs in fetal membranes, the effect is multiplied through the greater sensitivity to external forces within preterm membranes causing rupture, despite the thicker and more collagenous and GAG rich preterm membranes. This in turn results in the greater differences in mechanical and biochemical properties between non-ruptured and ruptured sites in preterm than term membranes that we observed. We show that calcium channel blockers exert their tocolytic effects through inhibition of aberrant mechanotransduction process in PPROM, even in the presence of persistent mechanical force.

Future work is needed to further define specific targets for more effective tocolysis agents. Given the potential significance of the heterogeneity between preterm and term membranes, further characterization of the interrelationship between mechanical and biochemical pathways in these tissues may provide insight to its role in term and preterm birth.

## Materials and methods

### Sample collection

Ethics approval was granted by the NHS Health Research Authority, West Midlands-Solihull Research Ethics Committee, UK (Reference 14/WM/1169). A total of 42 placental samples were collected following informed consent from study participants with singleton pregnancies: 22 from normal vaginal deliveries [10 preterm (33–36 weeks), 12 term (37–39 weeks)] and 20 from Caesarean sections (10 preterm, 10 term). Except for the external cyclic loading experiments, all membrane samples were from vaginal deliveries with spontaneously ruptured membranes, i.e. no prolonged rupture of membranes in either term or preterm deliveries. For the external cyclic loading experiments, we used membranes from term and preterm Caesarean sections. The specimens from vaginal delivery experienced labouring process, leading to pre-mechanical conditioning of the membrane, which are not suitable for cyclic loading experiments. Women with high risk pregnancies or chorioamnionitis were excluded from the study. The majority of the placentas were collected immediately after delivery. We stored 8 placentas in a designated fridge at 4 °C overnight but collected them within 12 h from delivery. The membranes did not freeze as a result of having been in the 4 °C fridge overnight. After sample collection, we conducted mechanical and Optical Coherence Topography tests immediately. We stored samples for Optical Coherence Elastography and some samples for immunostaining studies in a − 80 °C freezer for a short period due to time constraints. Therefore, the histochemistry, immunochemistry and Western blotting experiments were conducted with a mixture of fresh and frozen samples. We slowly thawed the samples on ice and performed live/dead staining analyses to assess the viability of the membranes prior to conducting further experiments. Above 90% viable cells were present in all samples used for the experiments. As we did not find any significant differences in cell viability between fresh and thawed samples in the live/dead staining analyses (Supplementary Fig. 1), we concluded that our collection and storage methods are unlikely to affect the collagen and cytokine levels. All experiments were performed in accordance with ethical standards in the 1964 Declaration of Helsinki and its later amendments.

### Displacement behaviour testing via ball indentation method

Specific to this study, we defined the fetal membranes from the edge of the tear zone as the ruptured site, while membranes distant from the tear zone (6–10 cm from rupture site dependent on size of the placenta or the rupture) was designated as from non-ruptured site (Fig. 1). Samples (approximately 25 mm in diameter) of the intact fetal membrane chorioamnion (CA), amnion alone (A) and chorion alone (C) from both ruptured and non-ruptured sites were used in the ball indentation test following established protocol in our laboratory, as previously described^19^. The ball indentation will acquire easily the membranes’ displacement behaviour which is the measurement of displacement change against time under the constant load. Briefly, membrane sample was placed between two transparent plastic rings (20 mm in diameter) which were held between two metal plates with a circular opening. The plates were tightened by multiple metal screws to reduce the risk of slippage as sample slippage is a possible source of experimental error^19^. This assembly was positioned in a petri dish with a plastic ball (76 mg, 2 mm in diameter) placed on the centre of the membrane serving as a spherical indenter to induce displacement by its gravity. The petri dish was placed in an incubator at 37 °C and 5% CO_2_ for a defined time period. The membranes were kept in PBS for the measurement. Images of the displacement were taken by a long focal distance microscope connected to a Charge Coupled Device (CCD) camera which was linked to the computer. The side view images of membrane displacement were used to quantify the mechanical behaviour of displacement. To quantify the displacement, image J software (IJ 1.46r, https://imagej.nih.gov/ij/) was used. The value of the displacement, i.e. the difference between initial (time zero) and final position after loading at the central location (the ball position) from image J software were expressed in pixels for convenience. We also performed preliminary studies on the mechanical properties of fetal membrane samples from vaginal delivery and Caesarean section at ruptured and non-ruptured sites using the ball indentation test. The results are presented in Supplementary Fig. 2.

### Optical coherence elastography

Quantitative measurement of the mechanical properties of each tissue sample was conducted using a novel OCE system built at the University of Dundee. The details of the system have been described previously^21^. Briefly, the OCE system consists of two main parts: vibration stimulation and signal detection. It employed a super-luminescent diode (SLD) as the light source, with a central wavelength of ~ 1310 nm and a bandwidth of ~ 110 nm. An objective lens of ~ 50 mm focal length was used to deliver the detection light onto the specimens and coupled the vibration signals into the phase sensitive optical coherence tomography for detection. The system provided an axial resolution of ~ 6.9 μm and a transverse resolution of ~ 11.7 μm in air. For the acquisition of a cross-sectional 2D structure and elastogram, the OCE probe beam stayed for 512 repeats during the A-line scan (one dimensional) at every spatial location sequentially within the B-scan (two dimensional, a total of 512 locations) mode while the actuator repeatedly fired the stimulus. Thus, a complete B scan consists of 512 × 512 A-scans. Before the scanning procedure, each tissue sample was defrosted from the dry ice and placed on 2% agar layer with a thickness of ~ 5 mm which was used as an elasticity reference^21^. Agar gels with defined concentration of 2% in cubic shape was formed and their modulus were measured by the average phase velocity, Eq. (1). The modulus of agar was 193 kPa with high reproducibility. Three B-scans were taken from different positions of each specimen. The scanning time for each specimen was approximately 3 min. During such short period of time, the specimens were assumed to have stayed hydrated. The raw structure and elastogram data sets were then processed by MATLAB R2015b (The MathWorks, Natick, MA, USA) for data processing to generate structure and elastogram frames for each B-scan. The OCE images were acquired once homogeneous vibration amplitude (no stress concentration) was achieved through pre-scanning monitoring while initiating the vibration on the samples.

### Optical coherence topography

Membrane thickness was measured using an OCT machine (Telesto II, Thorn lab, USA) with the centre wavelength at 1300 nm, which provides approximately 2 mm sample penetration, 13 µm lateral resolution and 5.5 µm axial resolution in air. Both term and preterm membranes at ruptured and non-ruptured sites were tested with 6 replicates per location.

### Histochemistry

Chorioamniotic membrane was fixed with 4% paraformaldehyde (PFA) and 4–6 µm sections were cut by a cryostat (Bright, UK). Pico Sirius Red staining were undertaken for the sections. Pico Sirius Red stain solution (0.1% w/v) was added and the samples were incubated for 1 h and washed with distilled water. Following this, the sections were washed twice with acidified water (0.5% acetic acid solution) and imaged. Total collagen content of the membranes was quantified by Hydroxyproline assay (Sigma-Aldrich, UK). 10 mg of tissue samples were weighted and homogenized first, then placed into PTFE topped glass vials. 100 µl of concentrated HCL (37%, 12 M) and 100 µl of deionized water were added for hydrolysis overnight (12–18 h) at 110 °C. Then, 20 µl of the hydrolysates were transferred to one well of a 96-well plate. Hydroxyproline standard was prepared by taking 0- 10 µl of 0.1 mg/ml stock standard solution and topped up to 20 µl by deionized water. The standard solutions and unknown samples were placed in an oven at 60 °C for drying. Following this 6 µl of freshly made concentrated Chloramine T solution and 94 µl of oxidation buffer mixture were added to each well and incubated for 5 min in room temperature followed by adding 100 µl of 4-(dimethylamino) benzaldehyde (DMAB) reagent (provided in the kit). The plate was incubated in an oven at 60 °C again for 90 min. 150 µl of the supernatants were transferred to a well of a new 96-well plate. The absorbance at 560 nm was read with a plate reader (BioTek Synergy II) for samples and standard.

Freshly made toluidine blue solution (0.1% w/v, pH 2.5) was used to detect sGAG in the samples. The toluidine blue solution was added onto the slides for 10–15 min’ incubation period at room temperature followed by washing with distilled water for three times prior to imaging. Quantitative measurement of total sGAG was conducted by 1, 9-dimethlymethylene blue (DMMB) assay^53^. For comparison between different groups, the same quantity of fetal membranes was digested by papain solution (125 μg/ml, pH 6.5). Next, 100 μl of each digested sample solutions were mixed with 200 μl of DMMB solution (1.6% w/v) for the reaction. Bovine tracheal chondroitin sulphate in distilled water (0–40 µg/ml) was used as the standard. The absorbance of the reacted solution was read by using a plate reader (Synergy II Biotech) at 530 nm immediately.

### Immunochemistry

Cryo-sections of the samples were fixed in 4% PFA for 10 min. To reduce background staining and tissue autofluorescence, some samples were pre-treated with Sudan black B in a concentration of 0.1% in 70% ethanol (negative control images shown in Supplementary Fig. 3). The sections were incubated with a primary antibody (1:100) overnight at 4 °C. After washing with PBS, the secondary antibody (1:200) was added and incubated for 1 h at room temperature. Subsequently, the slides were counterstained with DAPI. The primary antibodies tested included: MMP-9 (Santa Cruz Biotechnology), MMP-13 (Gene Tax), Fibronectin (Santa Cruz Biotechnology) and IL-1β (Santa Cruz Biotechnology). FITC conjugated secondary antibodies (Santa Cruz Biotechnology) were used to detect expressions of the aforementioned markers in the membranes. The fluorescence images were taken by Confocal Laser Scanning Microscope (CLSM, Olympus Fluoview FV 1200 with Fluoview software (4.1 version)). A minimum of triplicate slides per comparison group were tested along with negative control (without using primary antibodies). ImageJ software (IJ 1.46r, https://imagej.nih.gov/ij/) was used to semi-quantify the intensity of immunostaining images by randomly selecting three areas for each group.

### Western blotting

Tissue samples were homogenised by RIPA lysis buffer (Sigma, UK) prior to protein extraction. Protein concentrations were determined by bicinchoninic acid method^54^. 40 μg/lane of total protein was loaded in duplicates and separated on NuPAGE 4–12% Bis–Tris gels by SDS-PAGE. Separated proteins were subsequently electroblotted to a polyvinylidene fluoride membrane (Millipore Corp, USA). Each blot was incubated overnight at 4 °C with primary antibodies to proteins of interest in the samples (1:500) after blocking with 5% semi-skimmed milk in PBS-T buffer (PBS with 0.2% Tween 20) for 1 h. After briefly washing with PBS-T buffer, membranes were incubated with horseradish peroxidase conjugated secondary antibodies (1:1000) for 60 min. The membranes were reblotted with anti-GAPDH antibody (1:5000) as loading control. The same antibodies as those used in the immunochemistry assays were utilised.

### External cyclic loading experiments

The new external cyclic loading system consisted of two parts with the first part comprising of the same setting as the ball indentation method described above. Stainless steel balls (103 mg, 2 mm diameter) were used here instead of plastic balls to deform the membranes. The second part was a cyclically movable metal rod attached to a magnet at the end. While the magnetic rod moved toward and away from the metal ball (above the lid of the petri dish where the whole ball indentation system was placed), the metal ball was alternately lifted and dropped to the membranes (Fig. 5). The moving rod was driven by a see-saw motion rocker (Cole-Palmer, UK) through attachment of the rod to the rocker platform. The rocking speed was controlled at 30 rmp, which generated a cyclical loading frequency of 0.5 Hz.

The rocker and the ball indentation system were placed inside the incubator at 37 °C and 5% CO_2_ for the loading experiments. We used membranes close to the edge of placentas from Caesarean sections to conduct the experiments. Membranes were loaded and exposed to cyclical mechanical loading for 3 h with media in the petri dishes, followed by static incubation in media for 4 h. Nifedipine, a calcium channel blocker, was used to mitigate the loading effect. Samples were loaded with 10 µM and 30 µM nifedipine solutions in media during loading in comparison to those loaded with media only (control). After testing, the tissues were divided for immunostaining and Western blotting analyses to quantify the expression of MMP-9, MMP-13 and IL-1β.

### Statistical analysis

A minimum of 3 biological samples for each types of experiment and minimum of triplicate samples in each group were tested. All data were expressed as mean ± standard deviation (SD) and tested for normal distribution and homogeneity variance. Unpaired t-test was performed for comparison of means between two groups. Two-way analysis of variance (ANOVA) was performed to examine data on mechanical and biochemical testing among multiple groups. Turkey’s test was used for post hoc analysis. Statistical significance was expressed as *p*-value < 0.05. All statistical analyses were performed using Graphpad Prism 6 software.

## Supplementary Information

Supplementary Figures.
