# Supplementary material for: New approaches suggest term and preterm human fetal membranes may have distinct biomechanical properties
Source: Sci Rep. 2022 Mar 24;12:5109. doi: 10.1038/s41598-022-09005-2 (PMC8948223; doi:10.1038/s41598-022-09005-2)
Supplement: Supplementary file 1 — Supplementary Figures. [file 41598_2022_9005_MOESM1_ESM.pdf]

New approaches suggest term and preterm human fetal membranes have distinct biomechanical properties

Sudeshna Bhunia, Shaughn O'Brien, Yuting Ling, Zhihong Huang, Pensée Wu, Ying Yang

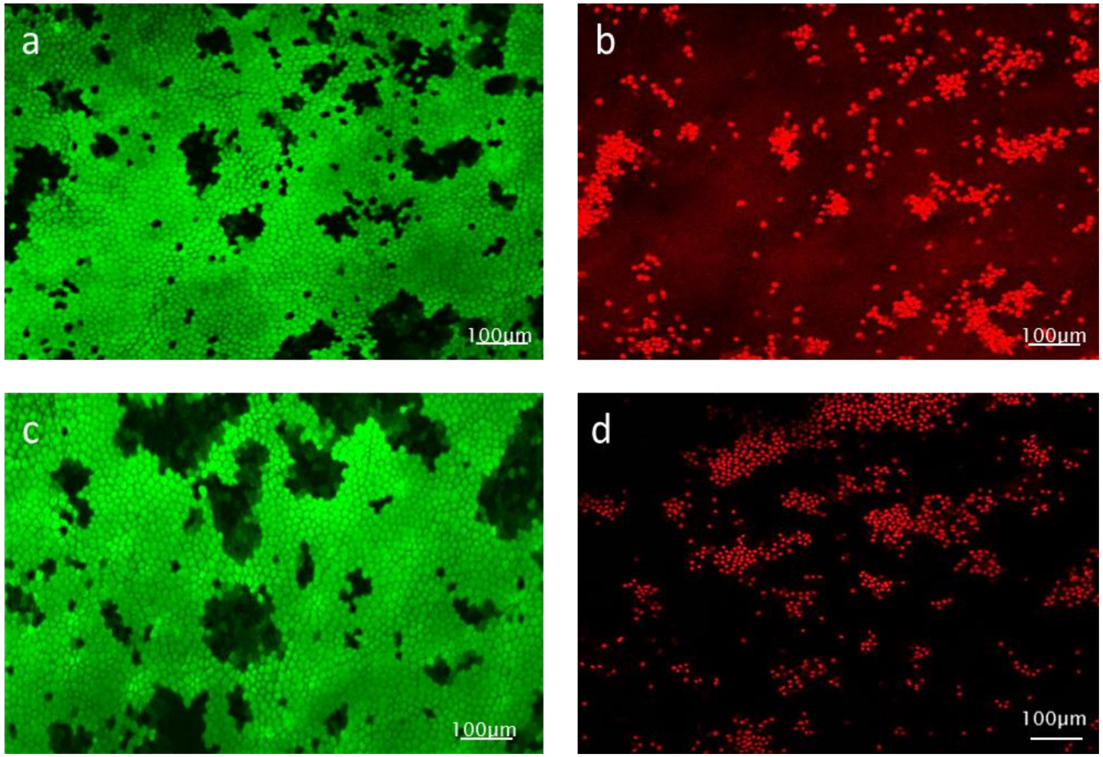

Figure 1. Live and dead staining of fetal membranes following 12 hour storage at 4°C and thawed fetal membranes following storage at -80°C. (a) Live cells of membranes at 4°C. (b) Dead cells of membranes at 4°C. (c) Live cells of membranes at -80°C. (d) Dead cells of membranes at -80°C. The Live cells stained green and the dead cells stained red in representative images of the membranes from 3 donors. Scale bar = 100 µm.

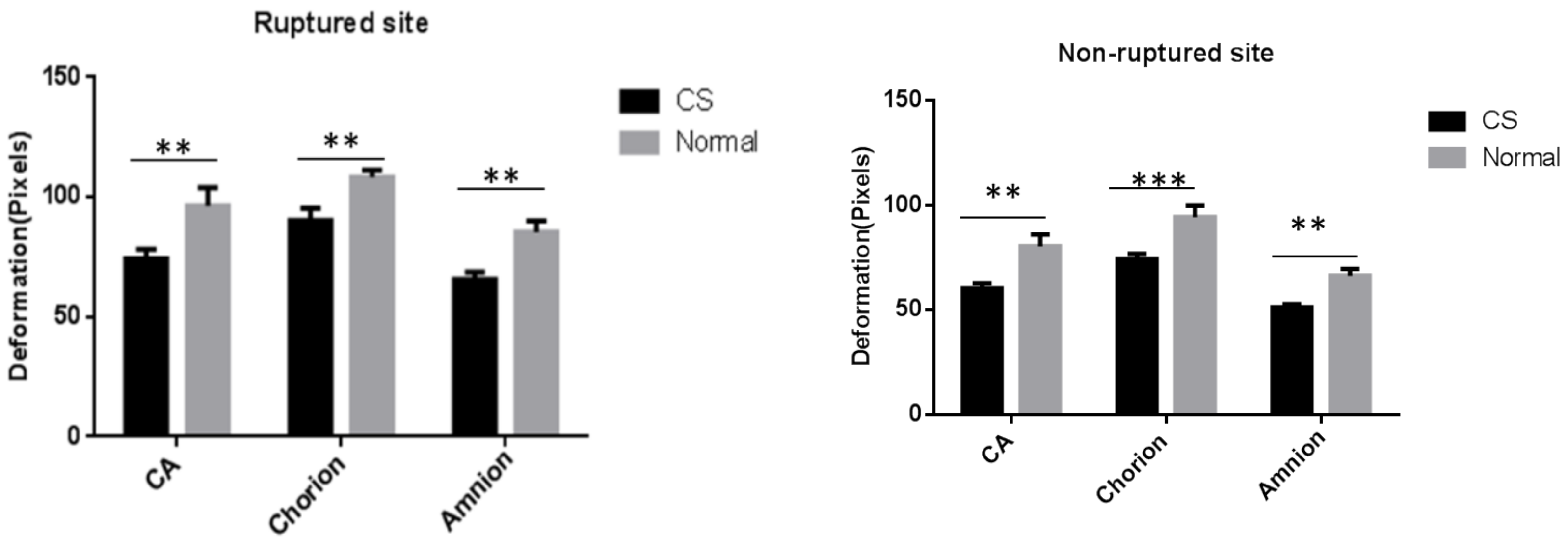

Figure 2. Preliminary study of mechanical properties of fetal membrane samples from vaginal delivery and Caesarean section by ball indentation test (n=4).

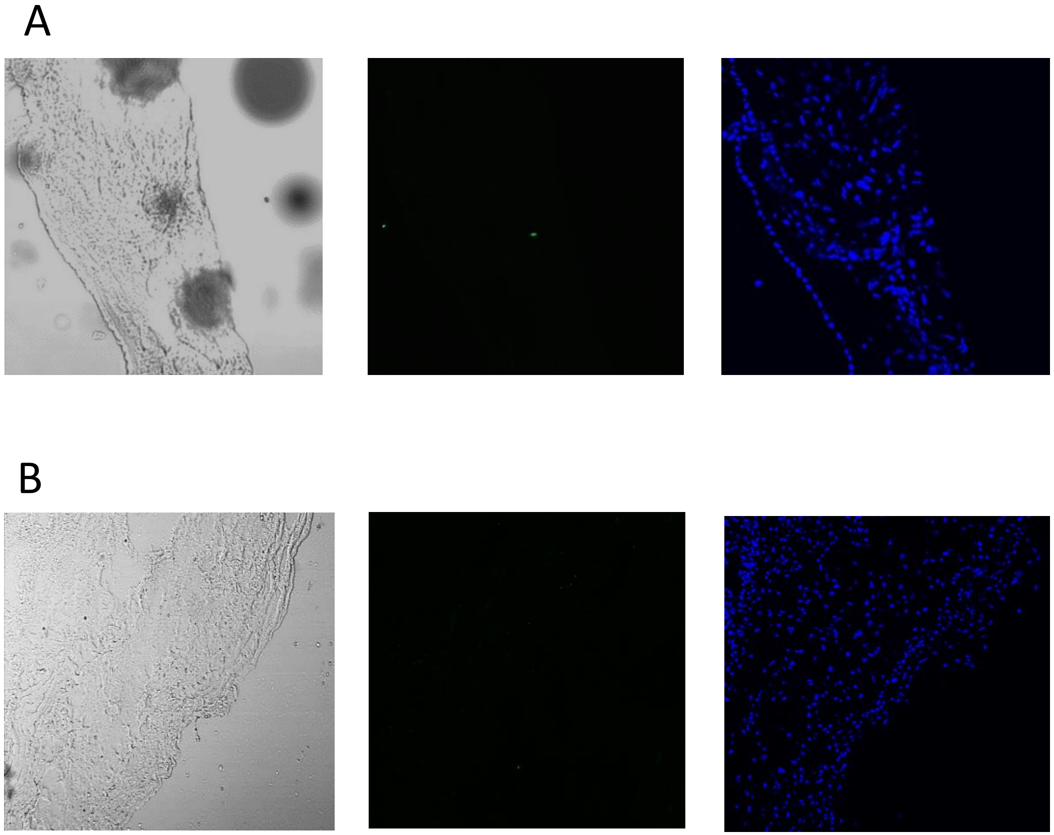

Figure 3. Negative control images for (A) MMP-13 (B) MMP-9 immunostaining. The samples were stained following all steps with in the absence of primary antibody incubation.

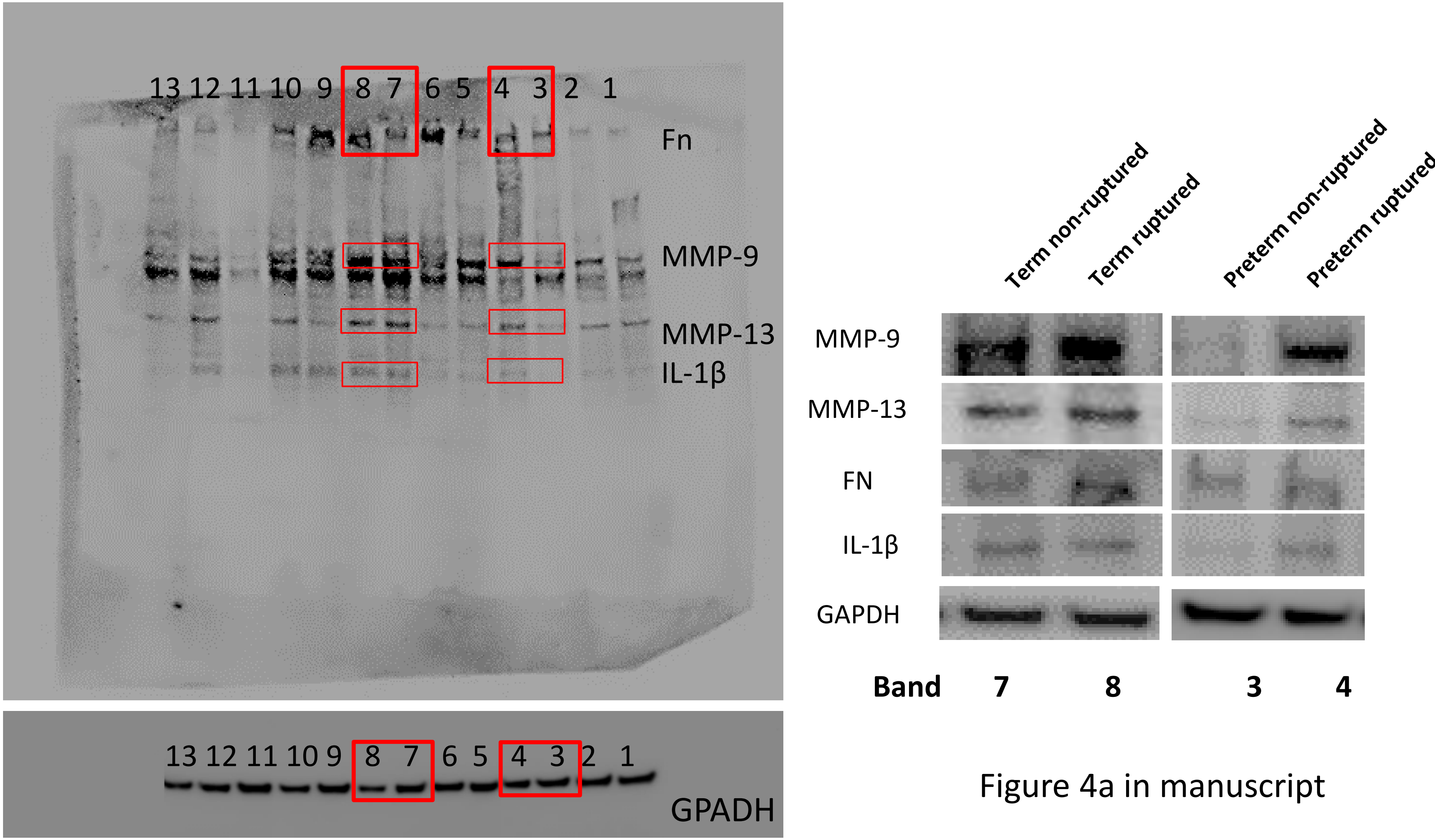

Figure 4 (I). Uncropped images of Western blots for Figure 4a. Band 3: preterm non-rupture; band 4: preterm ruptured; band 7: term non-ruptured; band 8: term ruptured.

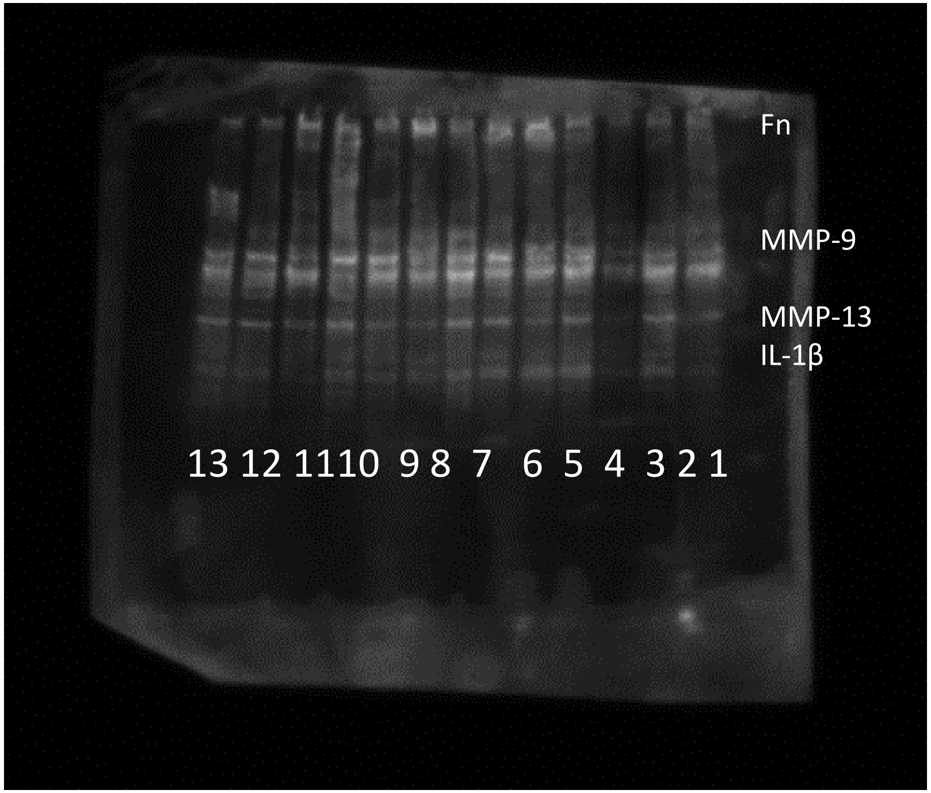

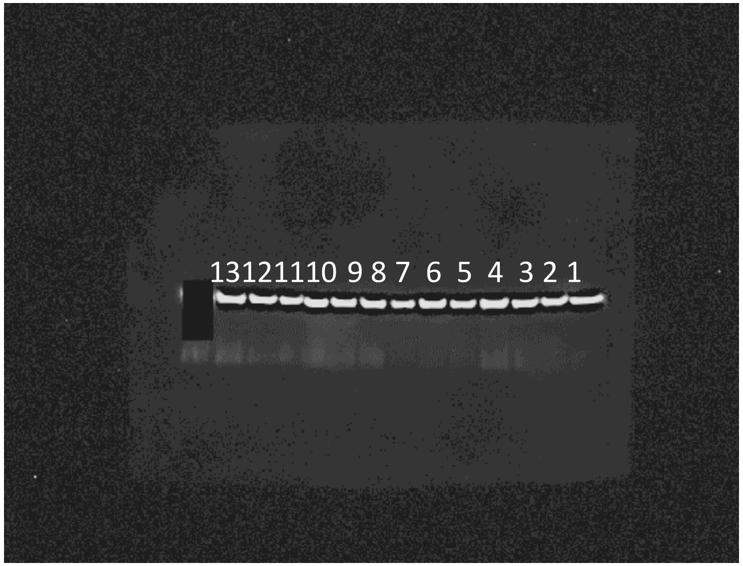

GAPDH

Figure 4 (II). Uncropped images of gels for Figure 4a with different exposure intensity. Band 3: preterm non-rupture; band 4: preterm ruptured; band 7: term non-ruptured; band 8: term ruptured.

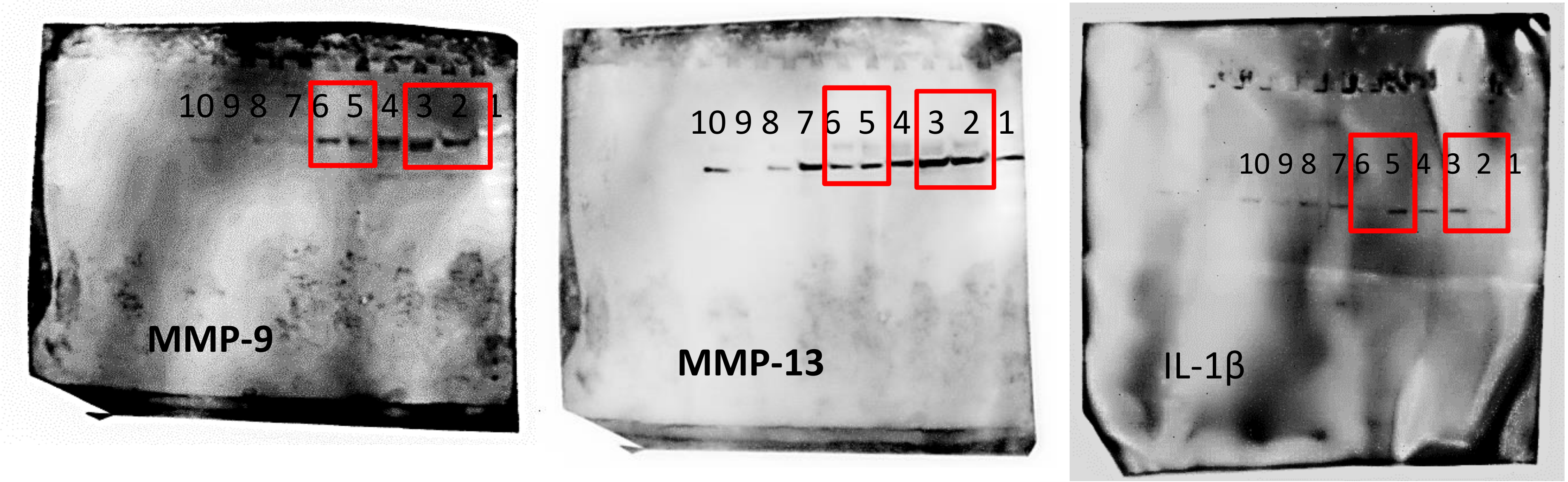

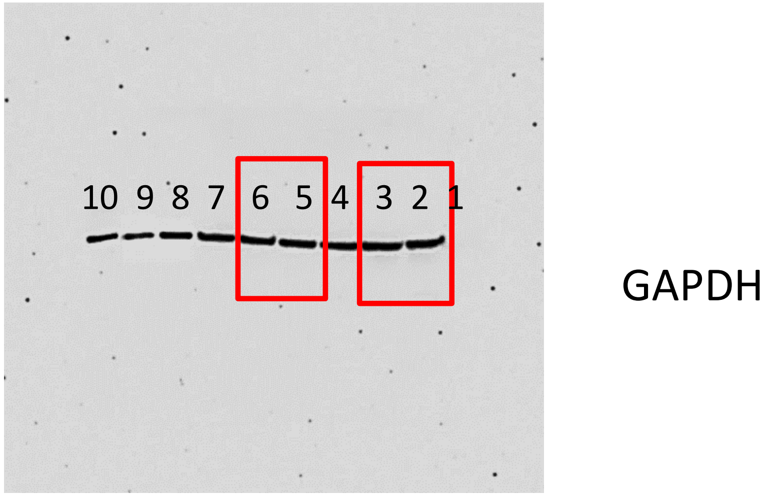

Figure 5 (I). Uncropped images of Western blots for Figure 7a. Band 2: term before stimulation; band 3: term after stimulation; band 5: preterm after stimulation; band 6: preterm before stimulation.

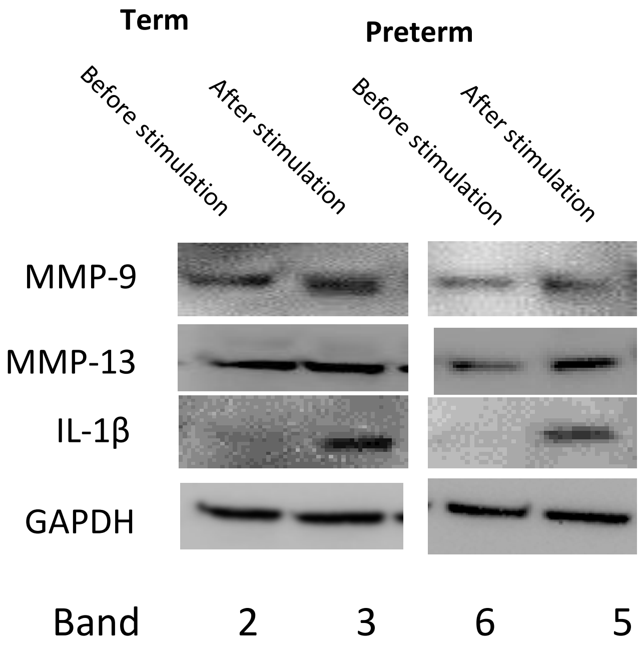

Figure 7a in manuscript

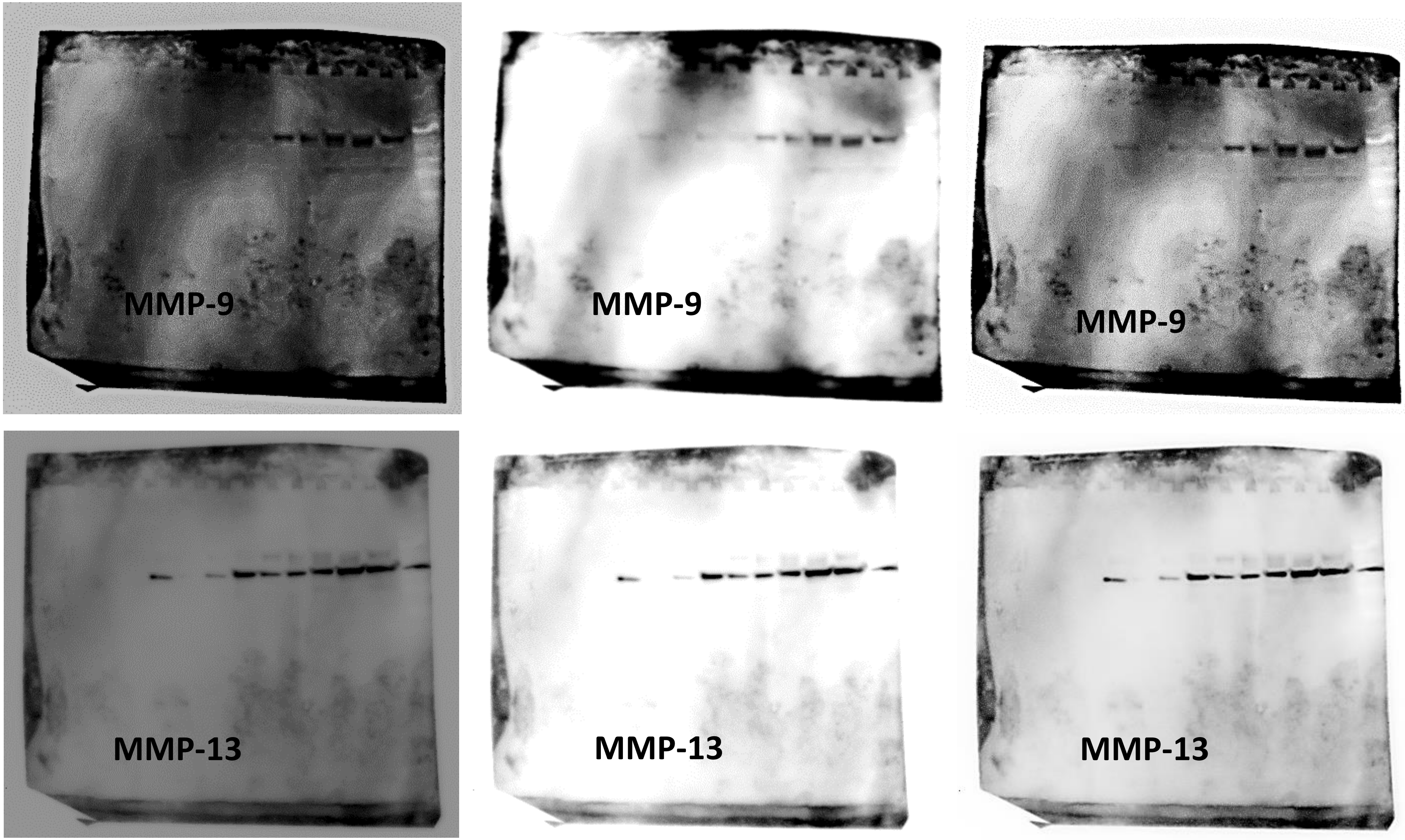

Figure 5 (II). Uncropped images of Western blots (MMP-9 and MMP-13) for Figure 7a with different exposure intensity and background. Left: longer; middle: shorter; right: proper exposure times.

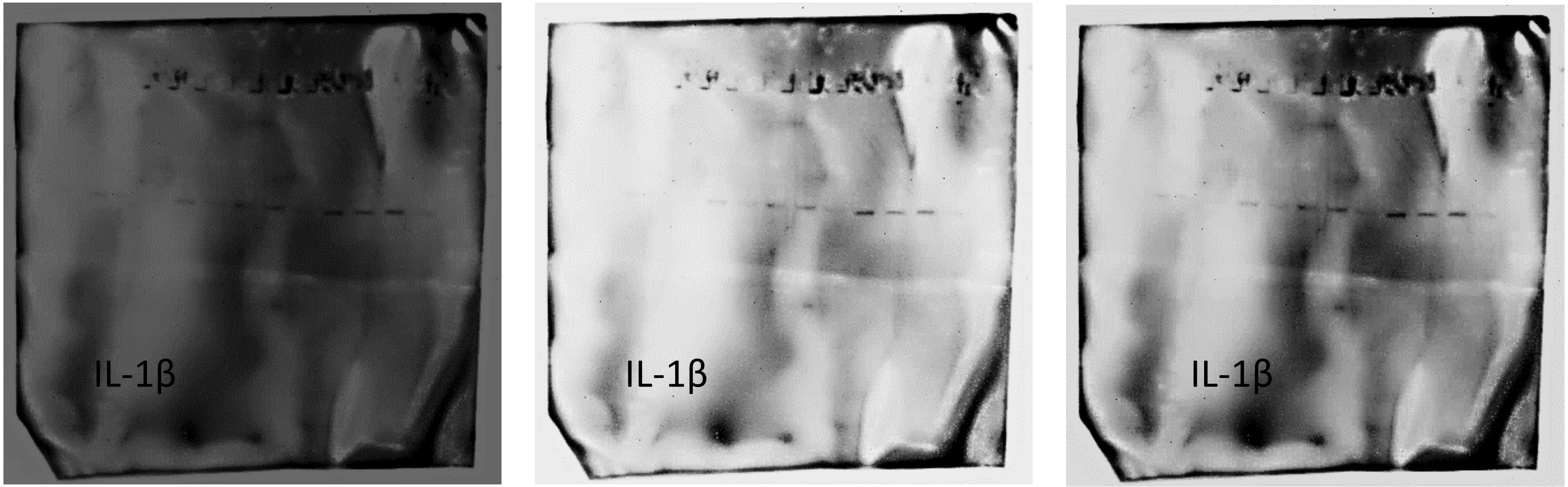

Figure 5 (III). Uncropped images of Western blots (IL-1 β) for Figure 7a with different exposure intensity and background. Left: longer; middle: shorter; right: proper exposure times.

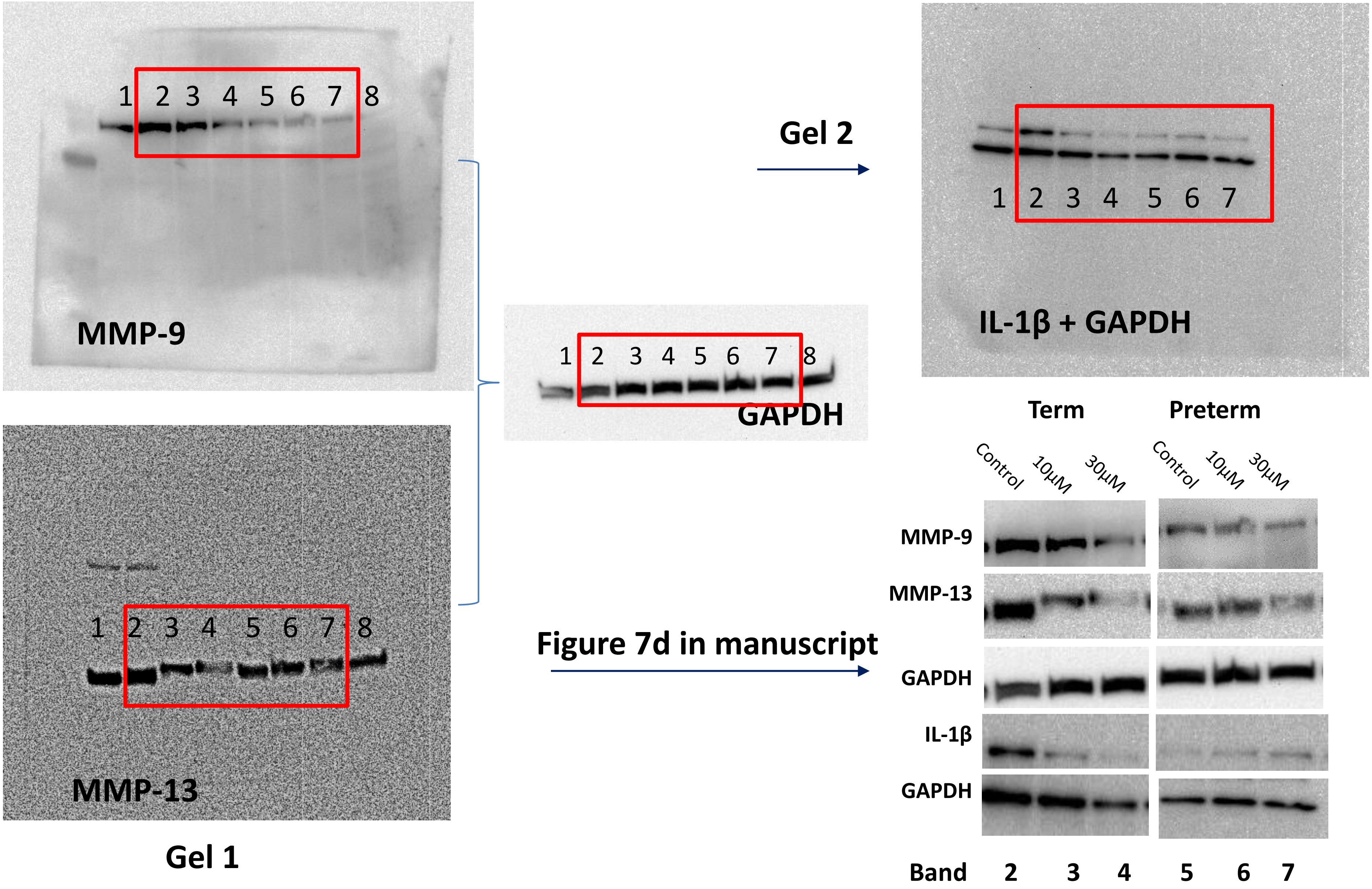

Figure 6 (I). Uncropped images of Western blots for Figure 7d**. Gel 1:** Band 2: term control; band 3: term load +10 µM nif; band 4: term load +30 µM nif; band 5: preterm control; band 6: preterm load +10 µM nif; band 7: preterm load 30 µM nif. **Gel 2:** Band 2: term control; band 3: term load +10 µM nif; band 4: term load +30 µM nif; band 5: preterm control; band 6: preterm load +10 µM nif; band 7: preterm load 30 µM nif.

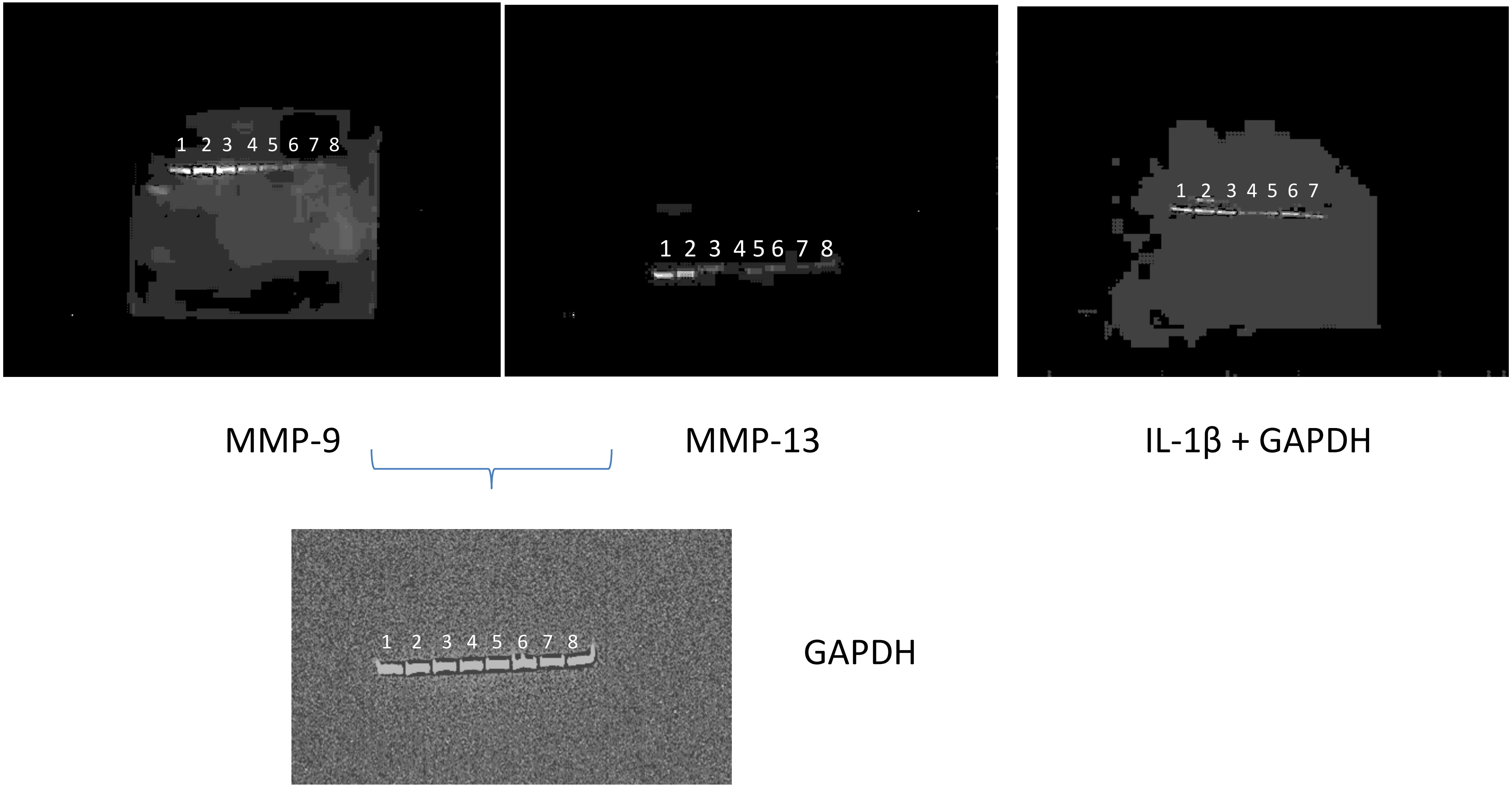

Figure 6 (II) Uncropped images of Western blots for Figure 7d with different exposure intensity and background. Band 2: term control; band 3: term load + 10 μM nif; band 4: term load + 30 μM nif; band 5: preterm control; band 6: preterm load + 10 μM nif; band 7: preterm load + 30 μM nif. (Two separate gels were run.)

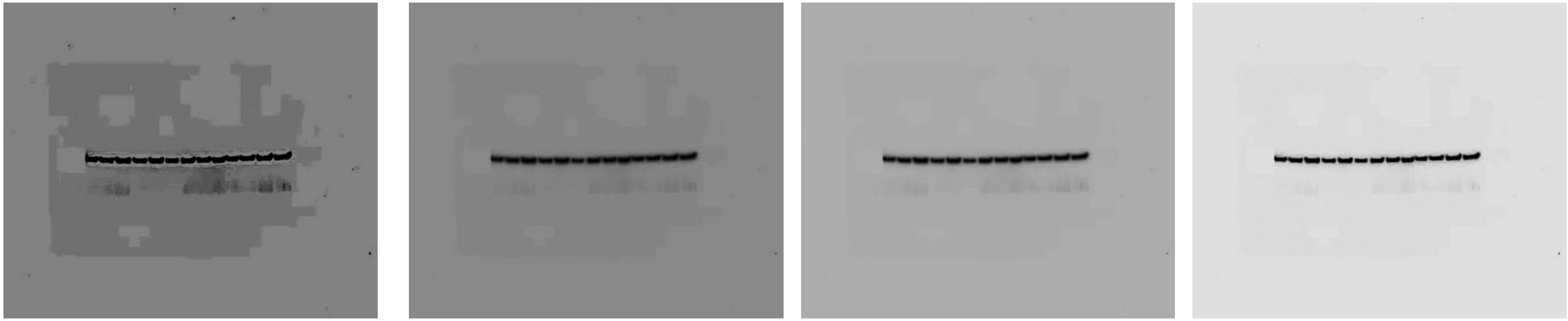

Figure 7 (I) GAPDH blot images showing original blot full-length with different exposure intensity for Figure 4a in the manuscript.

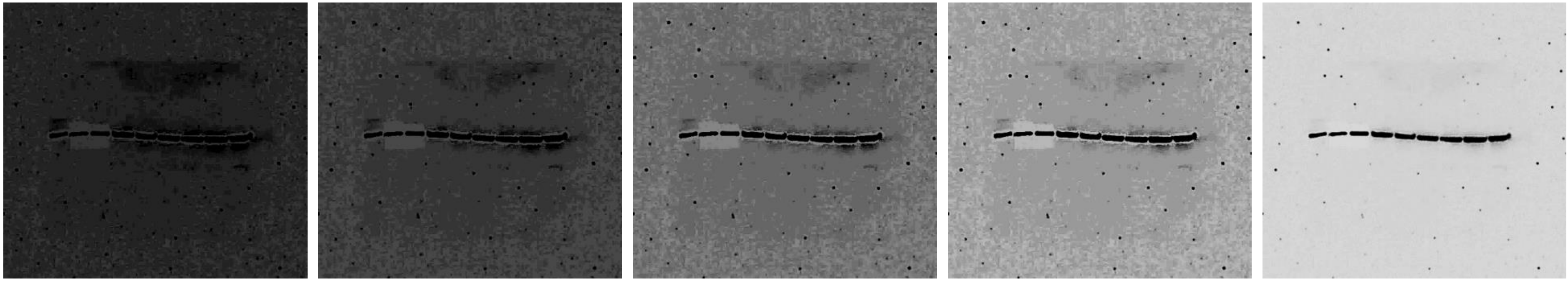

Figure 7 (II) GAPDH blot images showing original blot full-length with different exposure intensity for Figure 7a in the manuscript.

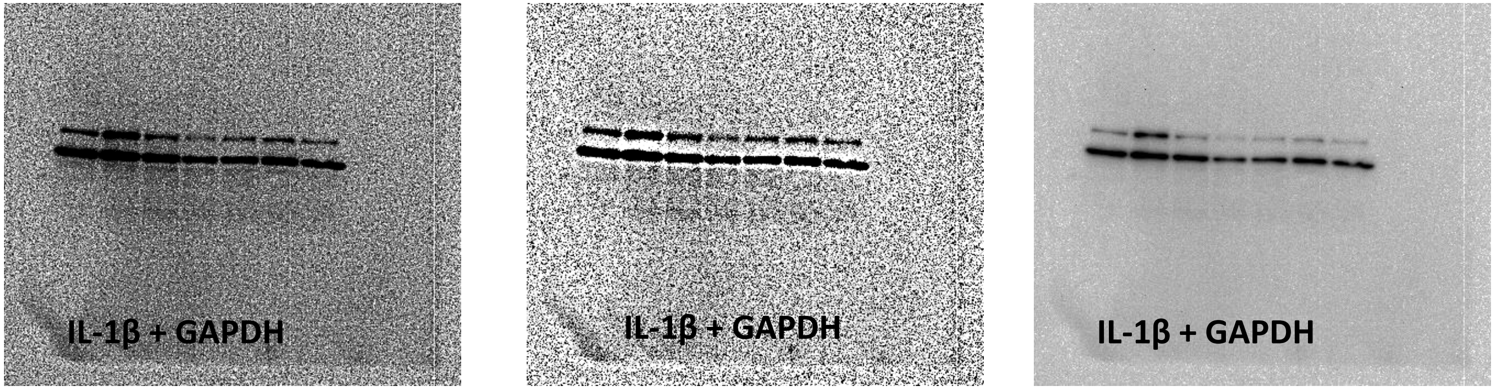

Figure 7 (III) GAPDH blot images showing original blot full-length with different exposure intensity for Figure 7d in the manuscript (gel 2).

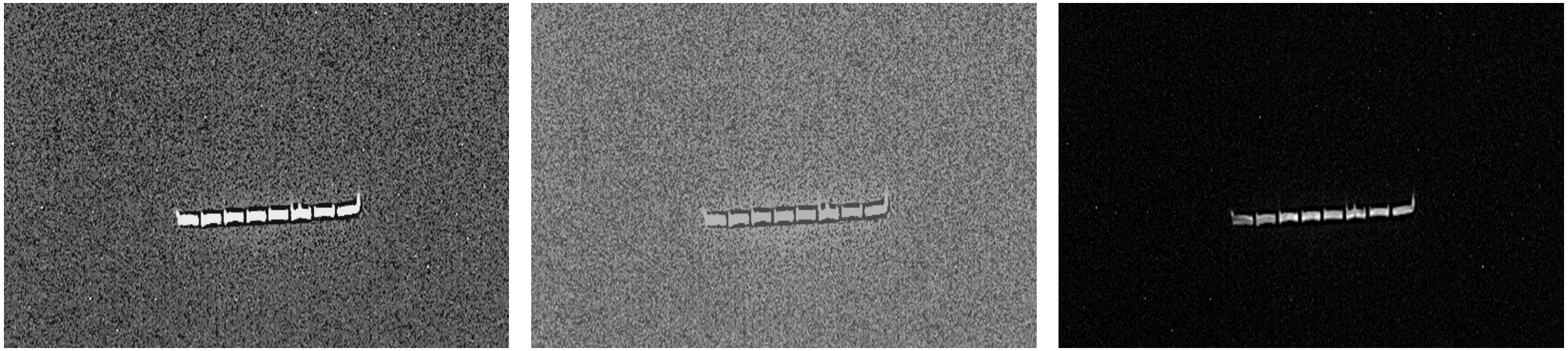

Figure 7 (IV) GAPDH blot images showing original blot full-length with different exposure intensity for Figure 7d in the manuscript (gel 1).
